# Caver Web 2.0: analysis of tunnels and ligand transport in dynamic ensembles of proteins

**DOI:** 10.1093/nar/gkaf399

**Published:** 2025-05-08

**Authors:** Sérgio M Marques, Simeon Borko, Ondrej Vavra, Jan Dvorsky, Petr Kohout, Petr Kabourek, Lukas Hejtmanek, Jiri Damborsky, David Bednar

**Affiliations:** Loschmidt Laboratories, Department of Experimental Biology and RECETOX, Faculty of Science, Masaryk University, 62500 Brno, Czech Republic; International Clinical Research Center, St. Anne’s University Hospital Brno, 65691 Brno, Czech Republic; Loschmidt Laboratories, Department of Experimental Biology and RECETOX, Faculty of Science, Masaryk University, 62500 Brno, Czech Republic; International Clinical Research Center, St. Anne’s University Hospital Brno, 65691 Brno, Czech Republic; Loschmidt Laboratories, Department of Experimental Biology and RECETOX, Faculty of Science, Masaryk University, 62500 Brno, Czech Republic; International Clinical Research Center, St. Anne’s University Hospital Brno, 65691 Brno, Czech Republic; Loschmidt Laboratories, Department of Experimental Biology and RECETOX, Faculty of Science, Masaryk University, 62500 Brno, Czech Republic; International Clinical Research Center, St. Anne’s University Hospital Brno, 65691 Brno, Czech Republic; Loschmidt Laboratories, Department of Experimental Biology and RECETOX, Faculty of Science, Masaryk University, 62500 Brno, Czech Republic; Loschmidt Laboratories, Department of Experimental Biology and RECETOX, Faculty of Science, Masaryk University, 62500 Brno, Czech Republic; International Clinical Research Center, St. Anne’s University Hospital Brno, 65691 Brno, Czech Republic; Institute of Computer Science, Masaryk University, 60200 Brno, Czech Republic; Loschmidt Laboratories, Department of Experimental Biology and RECETOX, Faculty of Science, Masaryk University, 62500 Brno, Czech Republic; International Clinical Research Center, St. Anne’s University Hospital Brno, 65691 Brno, Czech Republic; Loschmidt Laboratories, Department of Experimental Biology and RECETOX, Faculty of Science, Masaryk University, 62500 Brno, Czech Republic; International Clinical Research Center, St. Anne’s University Hospital Brno, 65691 Brno, Czech Republic

## Abstract

Enzymes with buried active sites utilize molecular tunnels to exchange substrates, products, and solvent molecules with the surface. These transport mechanisms are crucial for protein function and influence various properties. As proteins are inherently dynamic, their tunnels also vary structurally. Understanding these dynamics is essential for elucidating structure-function relationships, drug discovery, and bioengineering. Caver Web 2.0 is a user-friendly web server that retains all Caver Web 1.0 functionalities while introducing key improvements: (i) generation of dynamic ensembles via automated molecular dynamics with YASARA, (ii) analysis of dynamic tunnels with CAVER 3.0, (iii) prediction of ligand trajectories in multiple snapshots with CaverDock 1.2, and (iv) customizable ligand libraries for virtual screening. Users can assess protein flexibility, identify and characterize tunnels, and predict ligand trajectories and energy profiles in both static and dynamic structures. Additionally, the platform supports virtual screening with FDA/EMA-approved drugs and user-defined datasets. Caver Web 2.0 is a versatile tool for biological research, protein engineering, and drug discovery, aiding the identification of strong inhibitors or new substrates to bind to the active sites or tunnels, and supporting drug repurposing efforts. The server is freely accessible at https://loschmidt.chemi.muni.cz/caverweb.

## Graphical Abstract

**Graphical Abstract:**
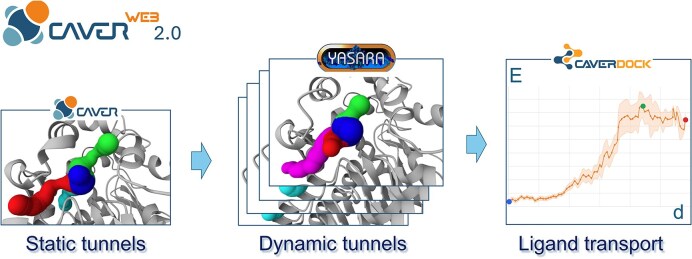

## Introduction

All proteins have features that may serve structural or functional purposes. Such is the case of voids, pockets, tunnels, or channels. We define a molecular tunnel as the structural pathway that leads from an occluded cavity in a biomolecule to its surface. When an enzyme has a buried active site, at least one tunnel is required for the entrance of the substrates, the release of products, or the exchange of solvent and co-factors. It is estimated that >64% of all enzymes contain molecular tunnels [1], which reveals how widespread these constructs are in nature. The physicochemical, geometric, and dynamic properties of these transport pathways play a crucial role in determining which ligands can and which cannot permeate them, thus affecting the enzyme function. The nature of the access tunnels can highly influence the catalytic activity, substrate specificity, enantioselectivity, and even protein stability [2, 3]. Therefore, an in-depth analysis of molecular tunnels can prove important to understanding or manipulating the natural properties of enzymes and other proteins. Such knowledge can be useful for the identification of bottlenecks in molecular transport, in explaining the substrate specificity of enzymes, for the discovery of selective inhibitors that bind the tunnels of biological targets, or even in the design of more efficient biocatalysts for specific substrates of interest. Multiple computational methods and strategies are available to expert users to attain accurate predictions on protein design and a deep understanding of molecular processes, such as ligand binding and transport [4, 5]. Inexperienced researchers can still find user-friendly tools to assist them in those tasks [6, 7], and Caver Web is one of them [8].

Caver Web 1.0 [7] is a web server that implements CAVER 3.03 [6], CaverDock 1.2 [8], and a virtual screening pipeline [9]. CAVER 3.03 [10] is a widely popular computational tool that enables the detection and characterization of molecular tunnels in proteins. CaverDock 1.2 [11] is a more recent tool that was designed to predict, in an inexpensive approximate manner, the trajectory and binding energy curves of a ligand travelling through an access pathway, and can be useful in protein engineering and other fields [3, 4, 12]. Virtual screening of FDA/EMA-approved drugs was implemented more recently [9]. These tools and methods have been validated previously. Caver Web analyses have been widely used to investigate access tunnel anatomy [13–17], improve enzyme activity [18, 19], explore the relationship between substrate specificity and substrate propensity to reach the active site [13, 20], assess ligand preference for different tunnels [13, 21], modelling stereochemistry [22], pathway engineering [23–25], pathway engineering [24, 25], perform virtual drug screening for validated disease-related targets [26], and even for guidance in advanced computational studies [27, 28].

Despite all the interesting applications available with the existing Caver Web platform, it comes with clear limitations. Like all biomolecules, proteins are intrinsically dynamic, and their flexibility and conformational plasticity are essential for their functioning. Their ability to adopt different conformational states enables them to bind the cognate ligands, perform enzymatic catalysis, be regulated by biological partners, and transduce molecular signals [29–32]. Therefore, molecular dynamics (MD) and related methods are increasingly used in biomolecular research. Protein flexibility can also have a significant impact on molecular tunnels, which can change their geometry considerably with respect to the ones found in the crystallographic, cryogenic electron microscopy structures or static computational models. It has been demonstrated that transport pathways may open and close over time, changing their topology, radius, length, and even the location of their bottlenecks, playing a role in determining substrate specificity and kinetics [3, 33]. Not surprisingly, also the transport of ligands is affected by the dynamic properties of the protein, and the binding of a ligand can often be correlated with the dynamic properties of the protein tunnels [2, 34, 35].

For the reasons described above, we decided to include MD in Caver Web 2.0, offering users the possibility to run short simulations and process the resulting ensembles in a user-friendly workflow. We want to emphasize that the purpose of this step is not to provide a complete overview of the conformational ensemble, which in some cases will require extensive sampling and advanced methods. The main goal here is to take flexibility into account and introduce some conformational variability with respect to the crystal structure. Caver Web 2.0 then automatically characterizes the tunnels over multiple snapshots and collects statistical parameters for the tunnel properties, as well as for the transport of ligands specified by the users. Caver Web 2.0 displays new capabilities with respect to the previous version, such as: (i) generation of dynamic ensembles with automatized MDs, (ii) analysis of dynamic tunnels in protein ensembles, (iii) analysis of ligand trajectories in multiple snapshots, (iv) customization of the ligand library for virtual screening, and (v) visualization of dynamic ensemble and ligand trajectories. These new features are expected to improve the quality of the analyses provided and enhance the success of functional studies. Here we describe the new implementations in Caver Web 2.0, together with other improvements in the web server.

## Materials and methods

Caver Web 2.0 was built using Next.js 14 (https://nextjs.org/), a React framework based on JavaScript. Among the software tools used in Caver Web, the main ones are CAVER 3.03 [10], which is used to compute the molecular tunnels in the input protein structure, and CaverDock 1.2 [11] to compute the trajectories and binding energy of the ligands travelling through the tunnels, inwards or outwards. YASARA 23.9.23 [36, 37] was introduced in the new Caver Web 2.0 to perform MD simulations and generate multiple snapshots to describe the dynamic ensemble. These tools were incorporated into a robust automatized workflow, which is described in the following section.

### Workflow and novel features

**Figure 1.**
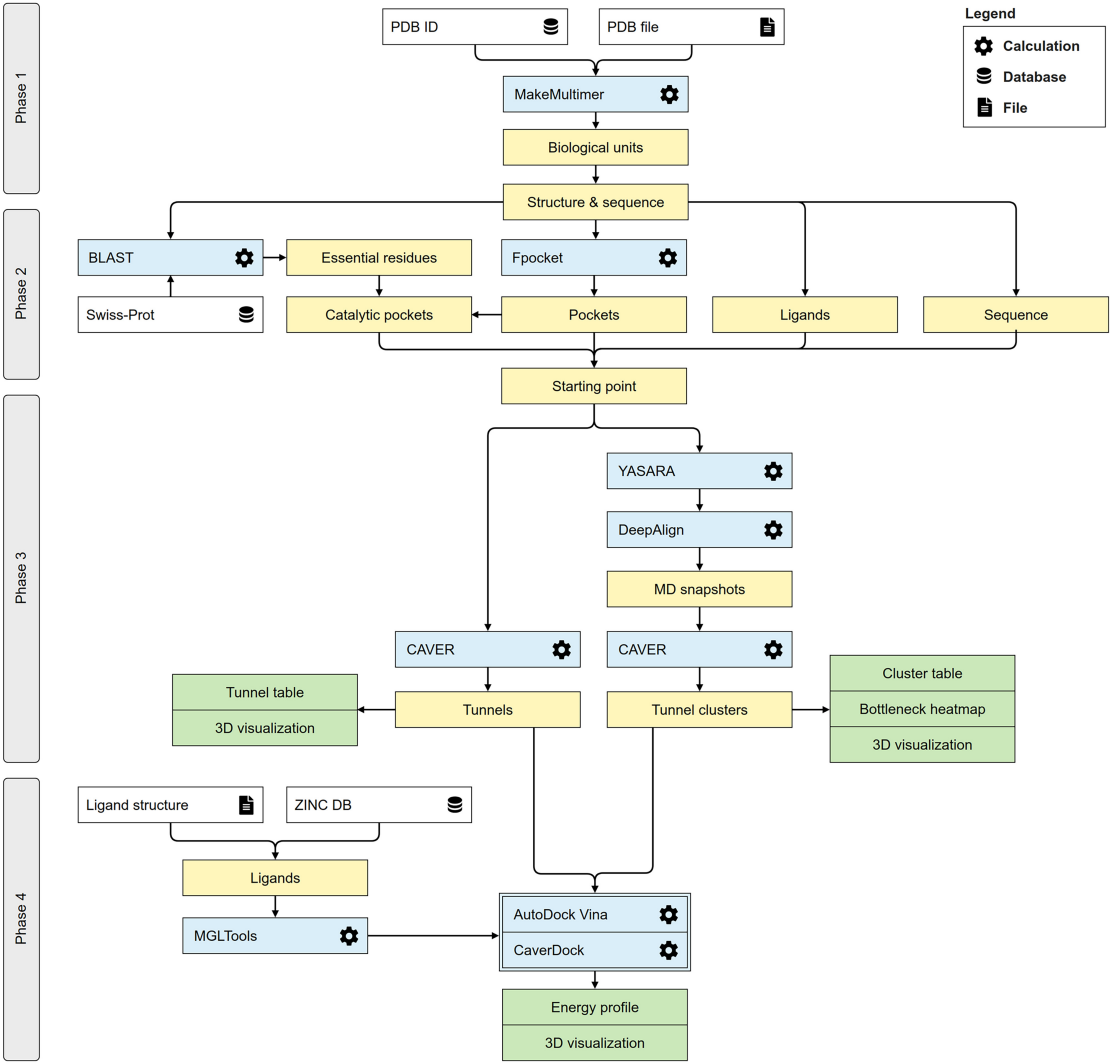
Schematic workflow diagram Caver Web 2.0. The process consists of four phases: (i) structure pre-treatment, (ii) specification of the starting point, (iii) identification of protein tunnels, with the option to run an MD simulation beforehand, and (iv) analysis of ligand binding and transport.

The workflow chart describing the implementation in Caver Web 2.0 is represented in Fig. 1. A large part of it is common to the Caver Web 1.0 workflow, which was previously described in detail by Stourac *et al.* [8]. In the first phase, the PDB file of the biomolecule is obtained from the Protein Data Bank [38] or uploaded by the user, and the biological unit is built by the *MakeMultimer.py* script, as described before [8]. In the second phase, the SwissProt database is searched using BLAST with the requirement of 30% sequence identity, to identify the essential residues. The pockets in the structure are computed by Fpocket 2 [39] to predict the most likely binding pockets (ranked by their relevance) and their druggability scores. The catalytic residues are matched with the calculated pockets to identify those that contain at least one catalytic residue. In the third phase, if the user is analysing a static structure, CAVER 3.03 calculates the tunnels directly on the input structure. It will construct additively weighted Voronoi diagrams [40] to identify all the pathways connecting the previously defined starting point to the surface, with a radius equal to or larger than a specified minimum probe sphere. The detected tunnels are listed with their geometric properties, and the 3D visualization of tunnels and protein is implemented with the Mol* 4.9.0 viewer [41].

Caver Web 2.0 can run an MD simulation before the tunnel analysis, and this is one of the main novelties of this web server (Fig. 1). YASARA 23.9.23 [36, 37] will be run with macro scripts to: (i) prepare the system and topology, (ii) run the MD simulation, and (iii) post-process the simulation and prepare the analysis report. In the first stage, the protein is protonated at the specified pH, a cubic box of water molecules with edges at least 10 Å away from the solute is added, and ions are added to neutralize the system and reach 0.9% NaCl (physiological conditions). Prosthetic groups or co-factors are self-parametrized and the protein is described with the AMBER14SB force field [42], which is adapted by YASARA to the YAMBER equivalent [43]. YASARA 23.9.23 first performs energy minimization to remove steric clashes and optimize the system. The MD simulation is then performed in the isothermal-isobaric ensemble (NPT), using a time step of 2.5 fs. Long-range electrostatic interactions are handled by the Particle-Mesh Ewald method with a cutoff of 10 Å. Periodic boundary conditions are applied to maintain system continuity. Pressure control is applied using a solvent-based approach in YASARA 23.9.23, where the system monitors the water molecule density and adjusts the pressure accordingly to maintain a stable environment. [37]. Due to resource constraints, the maximum simulation time is currently limited to tens of nanoseconds. YASARA 23.9.23 then processes the resulting snapshots to remove waters and ions and save them in PDB format. Finally, it analyses the MD simulation and creates a detailed report with the time evolution of important properties. The snapshots representing the dynamic ensemble are aligned with DeepAlign [44] to superimpose the Cα atoms of the protein. CAVER 3.03 is then used to calculate the tunnels in all the snapshots and generate tunnel clusters in the context of the dynamic ensemble, with the same tuneable parameters as for the analysis of a static structure. The tunnels identified in the snapshots are clustered using the Murtagh average link clustering method [40, 45], with a specified clustering threshold. This threshold ensures that the average distance between tunnels within a cluster is smaller than the defined threshold value (default: 3.5 Å). This approach groups equivalent tunnels detected across different snapshots into a single cluster. Changing this parameter will impact the final number of tunnels: increasing it will lead to merging more tunnels in the same cluster (potentially losing resolution), and decreasing it will generate more clusters (potentially leading to split equivalent tunnels). A complete description of all the CAVER parameters can be found in the User Guide (available at www.caver.cz).

In the fourth phase, the ligand transport analysis is performed on the single structure or on the multiple snapshots from the dynamic ensemble. The ligand files can be obtained from the IDSM database with the ChemWebRDF application [46], uploaded by the user, downloaded from the ZINC20 database [47], drawn with the JSME interactive molecular editor [48], and converted with Open Babel [49]. The ligands and protein are converted to PDBQT format using the *prepare_ligand4.py* and *prepare_receptor4.py* scripts from MGL Tools [50], adding the Gasteiger partial atomic charges or preserving the ones present in the input ligand files. CaverDock 1.2 [11] calculations are then performed with the selected ligands on the selected protein tunnels. The *Discretizer* module of CaverDock 1.2 [51] is used to slice the tunnel into discrete cross-sections, or discs (equally spaced by a certain *discretization delta*). CaverDock 1.2 then sequentially docks the ligand onto every tunnel disc, using the AutoDock Vina algorithm [52] with spatial restraints. This docking considers the ligand as fully flexible and the receptor as rigid. This docking considers the ligand as fully flexible and the receptor as rigid. This calculation results in a trajectory of the ligand inwards or outwards through the tunnel, and a corresponding energy profile (variation of the ligand-receptor binding energy along the trajectory). Two types of calculations can be performed with CaverDock 1.2: (i) employing only spatial restraints of the ligand to each disc (so-called *lower-bound* trajectory), or (ii) applying also rotational restrictions on the ligand to guarantee its nearly-continuous motion (*upper-bound* trajectory) [51]. The latter is computationally more demanding, highly stochastic, and therefore more uncertain, and it is more prone to failure if multiple attempts are unsuccessful to find an acceptable trajectory.

Caver Web 2.0 newly allows the visualization of each ligand trajectory in the Mol* visualizer, in the context of the protein and the respective tunnel. In the dynamic mode, CaverDock 1.2 calculates the trajectory of the ligand in every snapshot where the selected tunnel was found (snapshots with a closed tunnel are omitted). The energy profile is then formed by the average energy in each disc of the tunnel (i.e. the average over discs with the same sequential position from the tunnel starting point), and the standard error (SE) is calculated from the standard deviation (SD) of the energy and the number of energy points at the respective disc (*N*) as ${\rm SE} = {\rm SD}/\sqrt N$. This approach for considering protein flexibility has been validated before, and it has been shown to be more accurate in assessing the ligand transport energetics than using a single crystallographic structure [35].

The virtual screening is enabled in the static structure mode. Here, the user can simultaneously submit multiple ligands for molecular docking and ligand transportation assessment, using AutoDock Vina 1.1 and CaverDock 1.2, respectively. The user can select all FDA/EMA-approved drugs from the ZINC20 database, or specify a list of ligands of choice. The screening of FDA/EMA-approved drugs with Caver Web has been previously described in detail [9], and the data set is used without preselection (4380 compounds in total). The option to customize the ligand library is a novelty in Caver Web 2.0, and the list of ligands to be screened can be uploaded or defined as described above. The grid box for the docking can be specified using: (i) the starting point for the tunnel calculation as the box centre and a box size of 20 Å in each direction of space, or (ii) the centre of the pocket of interest as the box centre and the box size as the geometric limits pocket extended by 3 Å in each direction. CaverDock 1.2 calculations are prioritized based on the docking results, and only the 50-top compounds with the best binding energies are used for that step.

## Web server description

**Figure 2.**
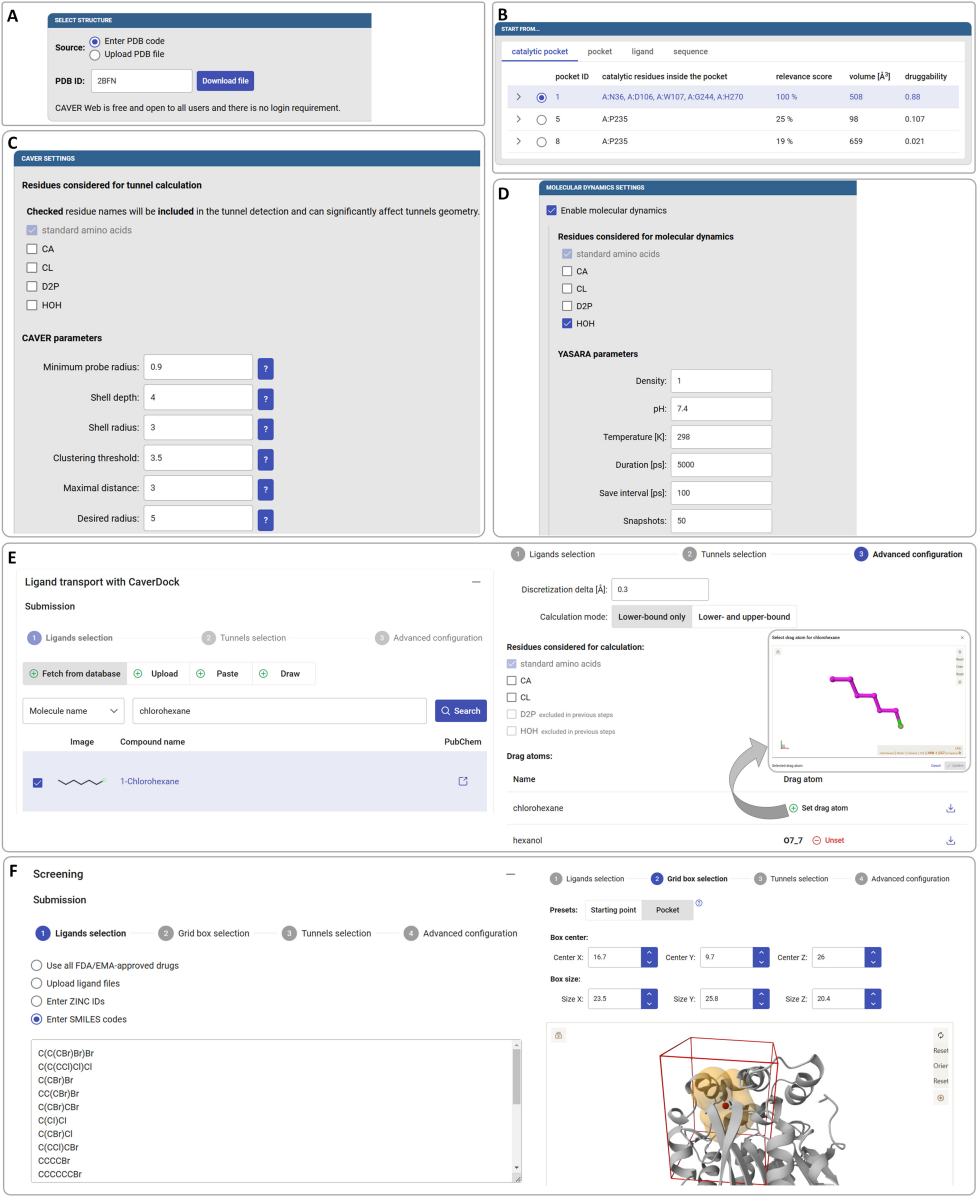
Inputs in the graphical user interface of Caver Web 2.0. The figure presents inputs for the enzyme haloalkane dehalogenase (HLD) LinB (PDB ID: 2BFN). (**A**) In the ‘Select structure’ panel, the user specifies the PDB ID or uploads the PDB file for their protein of interest. (**B**) The ‘Start from’ panel is used to define the starting point for the tunnel calculation, which can be done with four different approaches. (**C**) The ‘CAVER settings’ are used to specify the residues or molecules in the PDB to be considered and the parameters for the tunnel calculation. (**D**) The ‘Molecular dynamic settings’ allows the user to enable the MD calculation, define which molecules to include and specify several MD parameters. (**E**) The ‘Ligand transport with CaverDock – Submission’ section has three steps: (i) select the ligand(s) for analysis using one of four methods (left panel), (ii) specify the tunnel(s) to be used, and (iii) define advanced options, such as to specify the “drag atom” in a pop-up window (right panel). (**F**) The ‘Screening – Submission’ has four steps: (i) select the list of ligand(s) for analysis using one of four methods available (left panel), (ii) define the grid box for the molecular docking using one of two possible methods (right panel), (iii) specify the tunnel(s), and (iv) define advanced options.

**Figure 3.**
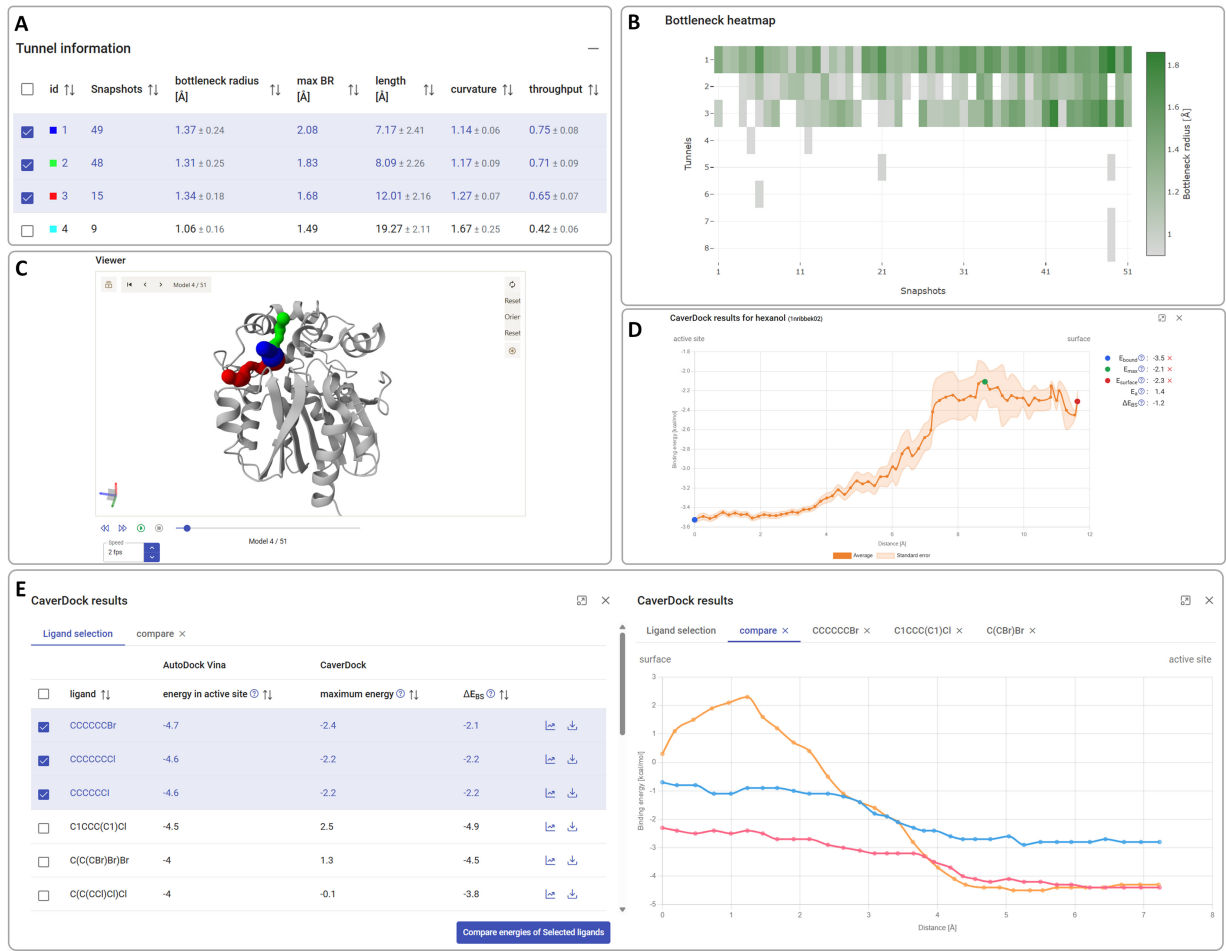
Outputs in the graphical user interface of Caver Web 2.0. The figure presents outputs for the enzyme HLD LinB (PDB ID: 2BFN). (**A**) The ‘Tunnel information’ panel provides a table with an overview of the detected tunnels (ranked by the throughput), in this case for dynamic tunnels with SDs. (**B**) The ‘Bottleneck heatmap’ panel illustrates the time-variation of the bottleneck radius for the different tunnels. (**C**) The ‘Viewer’ window displays the protein and the selected tunnels in an interactive 3D view. The colours of the tunnels are the same as for the respective entry IDs in the ‘Tunnel information’ table: tunnel 1 blue (middle), tunnel 2 green (upper), tunnel 3 red (lower). For the dynamic mode shown here, it contains playback buttons to visualize the changes in time. (**D**) The ‘CaverDock results’ pop-up window displays the energy profiles for the selected ligand along the tunnel with the SE for dynamic tunnels. (**E**) Screening results from docking using AutoDock Vina 1.1 and CaverDock 1.2 calculations for a series of ligands, in the form of a table (left) and a pop-up window with energy profiles for selected ligands (right).

The Caver Web 2.0 web server is organized in two major operational modes: (i) the single structure mode, and (ii) dynamic mode. The two modes differ mainly in the required inputs, the data presentation, and the type of calculations allowed. The single structure mode remains highly similar to Caver Web 1.0, and more details are provided in the respective publication [8]. Below we describe the inputs (Fig. 2) and outputs (Fig. 3) and highlight the differences between the two modes.

### Data input

#### Structure selection, enabling MD, and tunnel calculation

The user must define the 3D structure of the protein. This can be done by specifying the respective accession code from the Protein Data Bank [38] (PDB code) or uploading it as a file in PDB format (Fig. 2A). The biological unit is automatically detected, and the user is offered to use it. Subsequently, the starting point for the tunnel calculation is defined: (i) using the automatically determined catalytic pocket (Fig. 2B), (ii) any other pocket calculated with Fpocket, (iii) using one of the ligands or co-factors present in the PDB file, or (iv) a combination of amino acid residues in the sequence.

In the next step, the user can optionally provide a descriptive title of the job and an email address to be notified once the calculation is finished. The parameters for the tunnel calculation are defined in the ‘CAVER settings’ panel, where important parameters are presented with the respective definition (Fig. 2C). The user can modify the default values of important parameters that define the calculation of tunnels: (i) the minimum probe radius, (ii) shell depth and shell radius, (iii) clustering threshold, (iv) maximal distance and desired radius, described in the CAVER User Guide (available at www.caver.cz). The user may include specific molecules from the PDB, such as co-factors, and exclude any other molecules (such as ligands and ions). At this point, the user can enable MD (Fig. 2D) and modify several default parameters to run YASARA 23.9.23, namely: (i) molecules or co-factors to be included in the MD, (ii) the system density, pH, and temperature, (iii) duration of the simulation, and (iv) the time interval to save snapshots. After this, the job will run, and the results page will be displayed after it is finished. The content of this page will vary, depending on whether the user enabled MD or not (see below). The typical calculation time of tunnels in static structures is a few minutes. The MD can take between a few hours to one day to complete, depending on the length and size of the protein.

#### Ligand transport with CaverDock

After the protein tunnels are calculated, the user can optionally predict the trajectories of ligands travelling through the tunnels inwards or outwards. One or several ligands can be specified by: (i) searching for the chemical name in databases of small compounds (Fig. 2E, left), (ii) uploading a file compatible with Open Babel (PDB, MOL2, PDBQT, etc.), (iii) pasting the respective SMILES or ZINC codes, or (iv) drawing the 2D structure in the interactive molecular editor. The ligands obtained from databases are processed as retrieved. Ligands drawn in the editor are processed in their neutral form. If the user provides the ligand file in a specific protonation state, it will be preserved. Multiple protonation states are not considered in our pipeline. The automatically assigned name can be edited, and the trajectory direction (inward or outward) can be specified. Several ligands can be processed in a single submission round. Then, one or more tunnels for analysis must be defined. The advanced configuration allows: (i) changing the tunnel discretization step, (ii) enabling the upper-bound trajectory to be calculated (not by default), (iii) changing the molecules or co-factors to be included in the calculations, and (iv) specifying an atom of the ligand to be dragged along the tunnel (‘drag atom’, Fig. 2E, right). This feature constrains the atom to each cross-section of the tunnel instead of using the geometric centre of the ligand (default). This can be useful for long or flexible ligands, or when the user wants to specify the atom that will react in the active site (see supplementary Use Case 1 for more details). In dynamic mode, due to the existence of multiple tunnels in each cluster, with different lengths, the complexity of the task permits only the calculation of the outward direction and the lower-bound trajectories. After the submission, each ligand-tunnel pair will be run as an independent job. The ligand transport calculations typically take a few minutes to complete, but it depends on the length of the tunnel and the size of the ligand.

#### Virtual screening

This panel is restricted to the single structure mode due to its substantial computational resource requirements. It enables molecular docking and CaverDock 1.2 calculations to be performed simultaneously with multiple ligands. This function newly allows the user to personalize the list of ligands to be analysed. The complete set of FDA/EMA-approved drugs from the ZINC20 database can be selected. This feature offers a valuable resource for drug repurposing efforts targeting new disease-related proteins. Alternatively, the user can upload multiple ligand files, or specify a list of ZINC codes or SMILES codes (Fig. 2F, left). Then, two possibilities are available to define the centre and size of the grid box for the docking, either using the starting point in the tunnel calculation, or the previously selected pocket and its extended limits (Fig. 2F, right). If needed, those values can be fine-tuned manually. The tunnel to use in the CaverDock 1.2 calculations is then selected by the user. The virtual screening of FDA/EMA-approved drugs typically takes a few hours to complete. If the user submits only a few ligands, then this time can be shortened to a few minutes.

### Results output

#### Job information and tunnel analysis

After the tunnel calculation is completed, the results page is displayed. On the top-right corner of the page, the ‘Job information’ panel shows multiple details on the job, namely: the job ID, the title (if one was specified), the PDB ID or file name of the input structure, the starting time and status of the job. Below this, several buttons can be found to download useful files. The ‘Export to PyMOL’ button creates an archive file for download, which contains data and a script that, when run with PyMOL, generates a PyMOL session for detailed visualization of the protein structure and the tunnels (both in static and dynamic modes). In the static structure mode, the ‘Export to PyMOL’ also allows the user to include any of the subsequent calculations (ligand transport or screening) for visualization in PyMOL. The other buttons allow downloading raw data and report files from the CAVER 3.03 and pocket calculations. In dynamic mode, the user can find extra buttons to download the aligned snapshots from the MD simulation as PDB files, to visualize the detailed report from YASARA 23.9.23 on the MD analysis, or to download this report with the respective files.

The ‘Tunnel information’ panel displays a table with all the detected tunnels, with the respective ID and their geometric properties, namely: bottleneck radius, length, curvature, and throughput, defined and described previously [8, 10]. The tunnel profiles (radius vs. length) can be displayed and compared in a pop-up window. In dynamic mode, the snapshots (number of snapshots where the tunnel was found) and the maximum bottleneck radius (the largest bottleneck radius observed over all the snapshots) are also listed for each tunnel, and the tunnel geometric properties are represented by their average and SD over the ensemble (Fig. 3A). In static structure mode, the user can obtain extra details on every tunnel, such as an overview of the tunnel and its bottleneck, residue composition, coordinates, etc. In dynamic mode, the same detailed information can be found in the ‘CAVER data’ folder, available upon download. The tunnels can be selected and unselected for their visualization in the Viewer (see below).

In dynamic mode, the geometric properties of the tunnels are listed with their average values and standard deviation over the ensemble: bottleneck radius, maximum bottleneck radius (a single value), length, curvature, and throughput. The ‘Bottleneck heatmap’ panel displays the bottleneck radii of the tunnels over all the snapshots (Fig. 3B). It represents how the bottlenecks varied over time for the different tunnels, and it allows for interactively obtaining the details. The ‘Viewer’ panel shows the protein structure and the tunnels in a 3D visualization built with Mol*. In static mode, the protein pocket can also be visualized. In dynamic mode, the protein structure and tunnels are available for all the MD snapshots. In this case, playback buttons and a speed regulator control a movie visualization of the dynamic ensemble, and a slider allows quick navigation to specific snapshots (Fig. 3C).

#### Ligand transport with CaverDock

The ‘Results’ section of the ‘Ligand transport with CaverDock’ panel lists all the CaverDock 1.2 calculations submitted, with their status, name of the respective ligand, the dragged atom (if specified), tunnel ID, and direction. The user can visualize, in a pop-up window, the energy profile (binding energy vs. distance) of the ligand travelling along the tunnel for each job. The *lower-bound* trajectory is always displayed, but the *upper-bound* is also represented if requested during the job submission. Similarly to Caver Web 1.0 [8], the user can interactively annotate and store several energetic hallmarks (relevant energy points in the energy profile), such as: the energy in the active site (E_bound_), the energy maximum (E_max_), and the energy at the surface (E_surface)_. In single structure mode, the trajectory calculated by CaverDock 1.2 for each ligand can be visualized interactively in the 3D viewer panel. Here, the ligand is represented as sticks that move along the respective tunnel, represented by a transparent surface. The user can easily control the trajectory movie using the playback buttons available. For each calculation, the user can download the raw CaverDock 1.2 data, and generate a PyMOL session for a detailed visualization.

In dynamic mode, the user can also visualize the energy profile for each calculation. In this case, however, these profiles correspond to the average energy over the multiple trajectories in the different snapshots, and they are represented with the respective SEs (Fig. 3D). In this case, due to the complex nature of the data, the options are more limited, and it is not possible to interactively visualize the trajectories or generate PyMOL sessions.

#### Virtual screening

This panel is available only in single structure mode. The ‘Results’ section lists all the screening calculations submitted, with their status, job ID, tunnel ID, and direction of the CaverDock 1.2 trajectories. Detailed results are provided for each job in a pop-up window in the form of a table containing the 50-top ligands according to the docking scores. For these ligands are listed: the binding energy in the active site (from AutoDock Vina 1.1), the maximum energy in the tunnel (from CaverDock), and the difference between the energy at the surface and the energy minimum in the tunnel or active site (from CaverDock). The table can be sorted by ascending or descending order of any of those parameters. It is also possible to visualize and compare the CaverDock 1.2 energy profiles for any selected ligands in a separate pop-up window. For individual ligands, the user can manually assign the energetic hallmarks in the CaverDock 1.2 energy profile. The user can download the raw data containing the docking and CaverDock 1.2 calculations for all the 50 best ligands. A PyMOL session can also be generated for detailed visualization of the docked binding modes and the CaverDock 1.2 trajectories.

### Use cases

Caver Web 2.0 can be used to address various research questions, given that the enzyme or protein of interest has a buried binding site. In Use Cases 1 and 2 (Supplementary data) we guide users in performing and interpreting different types of calculations using Caver Web 2.0. We demonstrate the application of this web server in analysing the tunnels and ligand transport in HLDs, running virtual screening of different substrates, and illustrate the differences in the results from the traditional study with a single static structure and those from a dynamic ensemble.

#### Study of enzyme tunnels, ligand transport, and screening of substrates in static structure mode

HLDs catalyse the cleavage of carbon-halogen bonds in a large variety of halogenated compounds. These enzymes can be useful for the synthesis of fine chemicals, bioremediation of contaminated industrial sites or warfare chemicals, or biosensors [34, 53]. Among other factors, their substrate specificity can be related to the anatomy of their tunnels. In Use Case 1, we showcase the calculation of tunnels in LinB, DmmA, DhaA, DhlA, and DbjA (Supplementary data). Enzymes with shorter tunnels and wider bottlenecks have more open catalytic sites. HLDs with narrower tunnels convert smaller substrates with higher efficiency, while wider tunnels can accommodate larger molecules. For example, LinB prefers 1,2-dibromoethane, a small and hydrophilic substrate, while DmmA, with a wider tunnel, prefers larger molecules like 4-bromobutanenitrile [53]. Some enzymes adapt to bulkier substrates via conformational changes, which now can be studied using MDs in Caver Web 2.0.

Ligand transport calculations with substrate 1-chlorohaxane and the respective product 1-hexanol through the tunnels of LinB indicate that tunnel 1 is the preferred pathway for both substrate entry and product release. This is supported by lower activation energies (E_a_) for chlorohexane entering and hexanol exiting through tunnel 1 compared to tunnel 3. The lower E_a_ suggests faster transport kinetics, while the more negative energy difference between the bound state and the surface (ΔE_BS_) for tunnel 1 indicates stronger ligand binding at the active site. Additionally, chlorohexane shows a higher attraction to the mouth of tunnel 1 than tunnel 3, reinforcing its role as the primary transport route.

In the virtual screening of twenty substrates towards LinB, we found that all of them could bind favourably in the active site. This illustrates how versatile LinB can be to accommodate a wide variety of substrates. Transport through the main tunnel 1 showed relatively low barriers, although some of the linear substrates revealed more thermodynamically favourable profiles than bulkier cyclic substrates, which is in agreement with experimental data [53].

#### Study of enzyme tunnels and ligand transport in dynamic mode

Crystallographic structures, despite their enormous importance for understanding biomolecules, can be biased by crystal packing and represent only a snapshot of the protein conformational landscape. Use Case 2 illustrates how the access tunnels can be affected by the conformational fluctuation in a dynamic ensemble of LinB (Supplementary data). We found that the two main tunnels opened more widely in the MD than in the crystal structure and were detected very frequently (≥94% of the snapshots). Tunnel 3 was observed more scarcely (29%), but showed a relatively high throughput, while the remaining tunnels were observed very rarely (Fig. 3A and B). This analysis suggests that, although tunnel 1 is clearly the most preferred, the three main tunnels of LinB could potentially be used for the transport of small molecules, which is in agreement with the literature [54]. As demonstrated here, the study of dynamic systems enriches the results obtained from the tunnel analysis with a degree of confidence and detail that is difficult to achieve with a single structure.

When studying ligand transport in a dynamic context, we observed smoothing of the energy profiles due to conformational diversity. Steric clashes that hinder ligand movement in one snapshot may be resolved in the next by natural amino acid re-positioning. As a result, the calculated energy barriers reflect the ligand’s trajectory more realistically. Additionally, SE calculations provide a measure of uncertainty in the binding energies in each part of the tunnel. This effect was demonstrated in the transport of hexanol, analysed in both the crystal structure of LinB and its dynamic ensemble (Supplementary data and Fig. 3D).

## Conclusions and outlook

Caver Web 2.0 is a web server aimed at the identification and analysis of molecular tunnels in protein structures in a user-friendly environment. It can provide a graphical user interface to CAVER 3.03 [10] and CaverDock 1.2 [11] software tools, which are widely used to compute protein tunnels and to study the ligand transportation through the tunnels, respectively. It also enables the virtual screening of libraries of compounds targeting the protein of interest, for which molecular docking and ligand transport analysis are performed. This feature can be beneficial for identifying potentially strong binders for drug discovery, including the virtual screening of FDA/EMA-approved drugs against new disease-related proteins for drug repurposing, to identify new substrates of an enzyme, or to predict substrate specificity. The web server is designed to guide the user in setting up the calculations and interpreting the results properly, providing descriptions and explanations of concepts in multiple instances.

Caver Web 2.0 has implemented multiple improvements in comparison to version 1.0. The new version is built on a React framework, which can provide a better experience for the user. More importantly, it tackles a serious limitation of the previous version, which relied on static structures only. By enabling short MD simulations in a simple and automatized manner, the non-expert users can attain deeper and more realistic knowledge of their proteins of interest. Hence, this feature provides insights on: (i) what are the flexible regions, (ii) how the protein can adjust its conformation in a more biologically meaningful environment (aqueous solution instead of the crystal state), (iii) how molecular tunnels can change and vary when taking protein flexibility into account, and (iv) how those conformational changes may affect the transport of ligands through those tunnels. The dynamical changes can easily be visualized on the web server with the interactive 3D viewer Mol*. The geometric properties of the tunnels and the energy profiles of the ligand transport are now presented with statistical information that was not possible using a single structure. The advanced options for ligand transport calculations now permit the specification of a ‘drag atom’ within the ligand, facilitating a more precise representation of the binding process of interest, e.g. guiding a specific atom or substituent toward the active site. Finally, the newly introduced capability to customize the ligand set for virtual screening is highly beneficial for various applications, including drug design. This feature enables users to efficiently obtain molecular docking and CaverDock 1.2 results for individual molecules or larger sets of ligands under study. At this stage, two important clarifications are necessary. First, the short MD simulation is not meant to exhaustively sample the protein conformational space. That would probably require much longer simulations, possibly enhanced sampling methods, and expert interpretation. The main goal is to generate a set of protein conformations in a more biologically relevant environment than the crystal lattice, to assess the impact of protein flexibility on tunnel geometry and ligand transport. Moreover, the study of disordered proteins is not recommended here, where a few nanoseconds are most likely not sufficient for sampling reasonably diverse conformations. Second, CaverDock is not a replacement for detailed (un)binding MD simulations or free-energy methods. As an approximate docking-based approach, it is well suited for rapid screening or rough estimation. Although its accuracy improves significantly when multiple protein snapshots are considered [35], the resulting energy profiles remain approximate and are best used for comparative purposes rather than absolute energetic interpretation.

Caver Web 2.0 can still be improved in several ways. One of them is to extend the capabilities of the web server with longer MDs or more extensive conformational sampling. Due to the high computational requirements, we currently can offer MD simulations in the maximum range up to a few tens of nanoseconds. For some systems, especially the large proteins, this time scale may be insufficient to explore a diverse conformational space. To address this limitation, we plan to improve those calculations with Graphics processing unit (GPU)-acceleration, which we expect will allow us to perform at least one order of magnitude longer MDs within the same runtime. Another approach to tackle this problem is to allow the user to upload snapshots from their own pre-calculated MD simulations. In this way, users can run MDs with their favourite software tools, including enhanced sampling or any other method, and then perform the tunnel calculation and ligand transportation analysis with Caver Web 2. Moreover, recent advancements in machine learning-based techniques are expected to enable the generation of reliable conformational ensembles, such as AlphaFlow [55], ESMFlow [56], or BioEmu [57]. Such tools will enable a much faster and more cost-effective exploration of the conformational space. We will implement into the Caver Web these state-of-the-art methods after careful validation. Recent developments in CaverDock 1.2 have been made to account for the protein flexibility in the ligand transport through molecular tunnels, namely by considering side-chain flexibility and applying robotic algorithms to the ligand transport (manuscript in preparation). Once these methods are validated, we plan to make them available in Caver Web as an alternative to the current implementation. Finally, the user experience can be further enriched by implementing interactivity in the calculation of tunnels within the same job. The addition of new features, such as the automatic introduction of mutations and assessing their impact on the protein tunnels directly, would extend the capabilities of Caver Web in the field of enzyme engineering.

## Supplementary Material

gkaf399_Supplemental_Files

## Data Availability

Caver Web 2.0 is freely available and accessible at https://loschmidt.chemi.muni.cz/caverweb.
