# Supplementary material for: Caver Web 2.0: analysis of tunnels and ligand transport in dynamic ensembles of proteins
Source: Nucleic Acids Res. 2025 May 8;53(W1):W132–42. doi: 10.1093/nar/gkaf399 (PMC12230698; doi:10.1093/nar/gkaf399)
Supplement: gkaf399_Supplemental_Files [file gkaf399_supplemental_files.zip › UseCase1_single_structure_mode_R1.docx]

**Use Case 1: Study of enzyme tunnels, ligand transport and screening of substrates in static structure mode**

**List of Content**

[1. Comparing the access tunnels in the crystal structures of haloalkane dehalogenases 2](#_Toc194167652)

[Selecting the protein structure 2](#_Toc194167653)

[Determining the tunnel starting point 3](#_Toc194167654)

[Setting up the CAVER parameters 4](#_Toc194167655)

[Analyzing the tunnels 6](#_Toc194167656)

[Collecting the data 11](#_Toc194167657)

[2. Binding and unbinding of ligands though the access tunnels of a haloalkane dehalogenase 13](#_Toc194167658)

[Selecting the ligand 13](#_Toc194167659)

[Selecting the tunnels 16](#_Toc194167660)

[Setting up advanced parameters 17](#_Toc194167661)

[Analyzing the results 19](#_Toc194167662)

[3. Virtual screening of substrates in the active site and tunnels of a haloalkane dehalogenase 23](#_Toc194167663)

[Selecting the ligands 23](#_Toc194167664)

[Selecting the grid box 24](#_Toc194167665)

[Selecting the tunnels 26](#_Toc194167666)

[Analyzing the data 27](#_Toc194167667)

[References 32](#_Toc194167668)

## **1. Comparing the access tunnels in the crystal structures of haloalkane dehalogenases**

Load pre-calculated job for static structure: job [pxtxpx](https://loschmidt.chemi.muni.cz/caverweb/job/pxtxpx/)

Haloalkane dehalogenases catalyze the cleavage of carbon-halogen bonds of many types of halogenated hydrocarbons. These enzymes are useful biocatalysts for the purification of industrial waste waters, decontamination of warfare chemicals and in biosensors as biorecognition agents (1, 2). Haloalkane dehalogenases are closely related and their catalytic residues are nearly the same, but their substrate preferences vary. In this section, we will use Caver Web 2.0 to calculate the access tunnels in a crystal structure of haloalkane dehalogenase LinB using CAVER 3.03 (3) and shed light on which tunnel is more likely to be used for the transport or substrates and products between the bulk solvent (near the surface) and the active site. When comparing with other enzymes, one may find relationships between the properties of the tunnels and the substrate specificity of the different enzymes.

The usage of Caver Web consists of several sequential stages:

- Selecting the protein structure
- Determining the tunnel starting point
- Setting up the CAVER parameters
- Analyzing the tunnels
- Collecting the data

### Selecting the protein structure

To use a protein structure from the PDB, select the option “Enter PDB code” and the PDB ID of your protein into the corresponding PDB ID field on the Caver Web front page and click on “Download file”. Alternatively, you can also upload your own pdb file. Insert “2BFN” to the PDB ID field and press “Download PDB file” button.


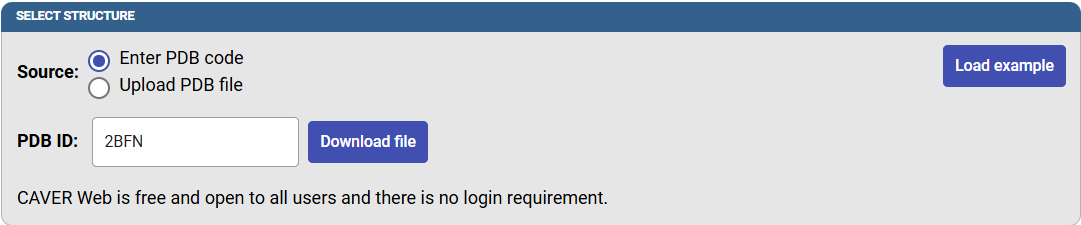


After this, the biological unit will be displayed according to the information contained in the PDB file. If you prefer to use the asymmetric unit instead, check this box. Click on “Next” to select the starting point for the tunnel calculation.


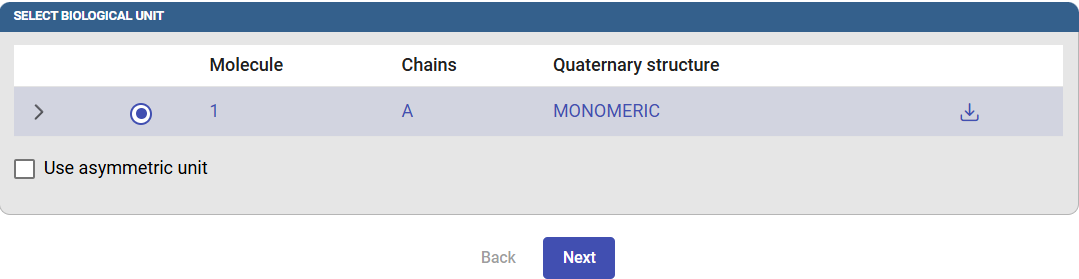


### Determining the tunnel starting point

Caver Web offers multiple options for selecting the tunnel starting point, which is typically located inside the active site. You can select it from the catalytic site information extracted from UniProt, from the calculated pockets, using the ligands present in the pdb structure or selecting residues from the sequence. Caver Web also provides visualization of the protein structure to aid the selection of the starting point. When entering the tunnel starting point phase, Caver Web will automatically detects the pockets, which might take a while if your protein is large (>500 residues).

In this tutorial we will use the catalytic pocket option. Click on the “catalytic pocket” tab to see the details of the found pockets. You can see in the table the predicted catalytic residues for pocket ID #1: A:N36, A:D106, A:W107, A:G244, A:H270, the volume and druggability score of the respective pocket. The druggability score tells on scale from 0 to 1 how much the pocket resembles the small molecule binding pockets. Select pocket #1 by clicking on the circle at the start of its row.


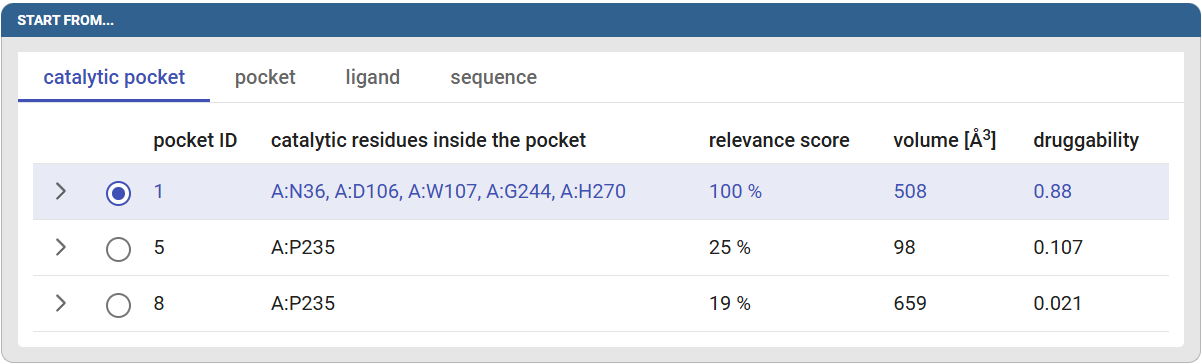


The pocket will be represented in the visualization pane below as a transparent orange surface with a red sphere at its center. This sphere will be used as the starting point for the tunnel calculation.

You can check the “Enable manual fine tuning” and manually change the Cartesian coordinates of the starting point. Click on “Next” to define the remaining Caver parameters for the tunnel calculation.


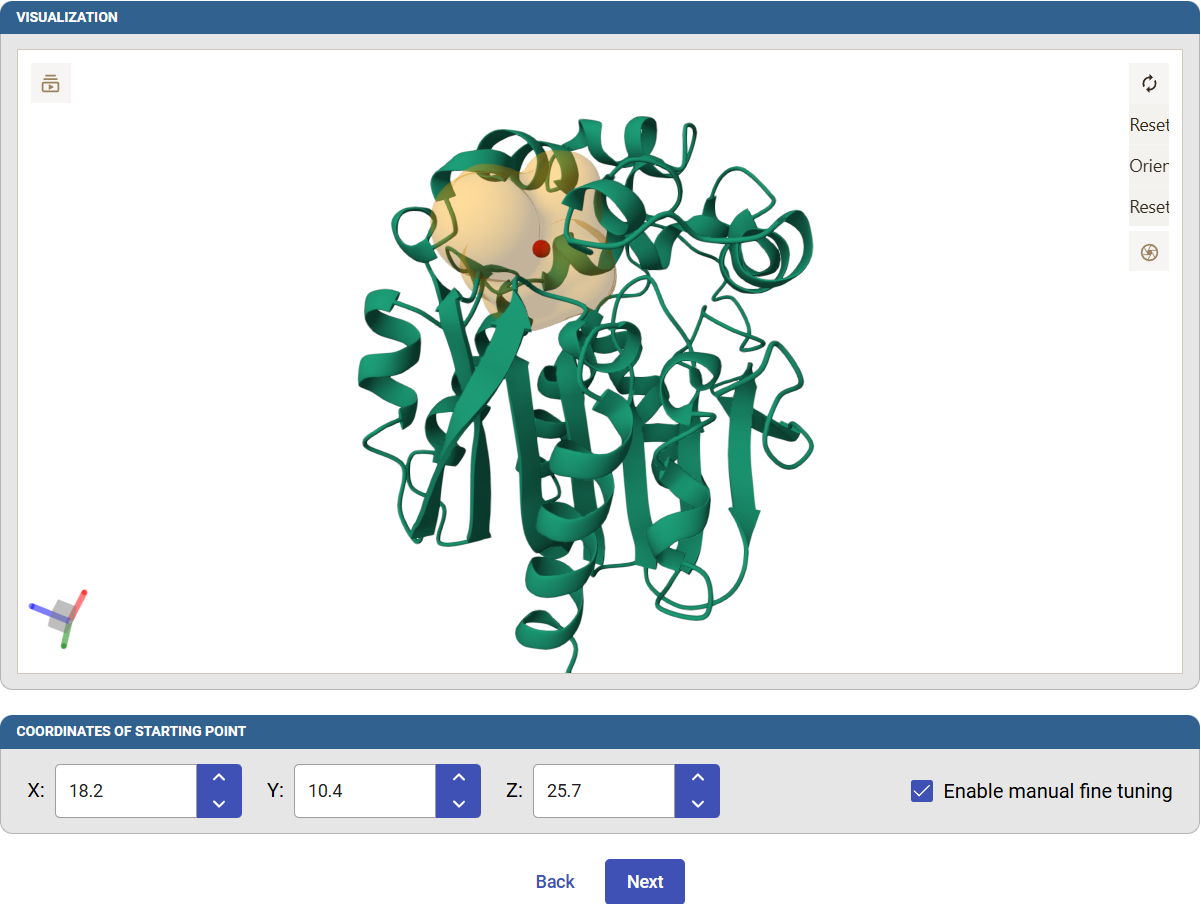


### Setting up the CAVER parameters

First, you can include a description title to your job and an email address to be notified once the calculations are finished. A typical job of tunnels calculation takes only a few minutes.

Caver Web allows you to run molecular dynamics with your system before the calculation of tunnels, but we will illustrate this option in a different example case, so do not check that box.


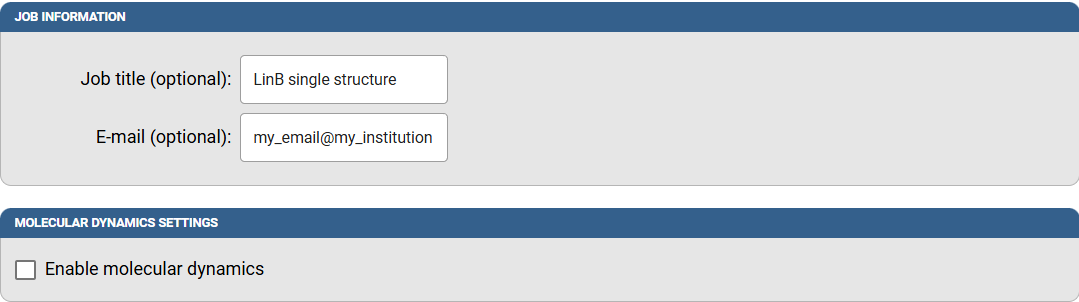


You are prompted to select which parts of the PDB structure to be included or excluded during the tunnel calculation, such as co-factors, waters or co-crystalized ligands. For example, sometimes co-factor molecule in the active site can be included in the calculation, but water molecules most likely not (since they would block the tunnels that you want to compute).

The CAVER parameters determine how the tunnels are calculated. You will find more information about the individual settings by clicking the “?” icon next to them. There are also buttons to reset the values to defaults. The default settings have been determined through extensive testing and they should provide users with reliable data in most cases. The most crucial parameter is the “Minimum probe radius”, since it defines how narrow the tunnels can be. Standard default settings will be used throughout this tutorial. If you changed some values, click on “Reset values”. Finally, click on “Run job”.


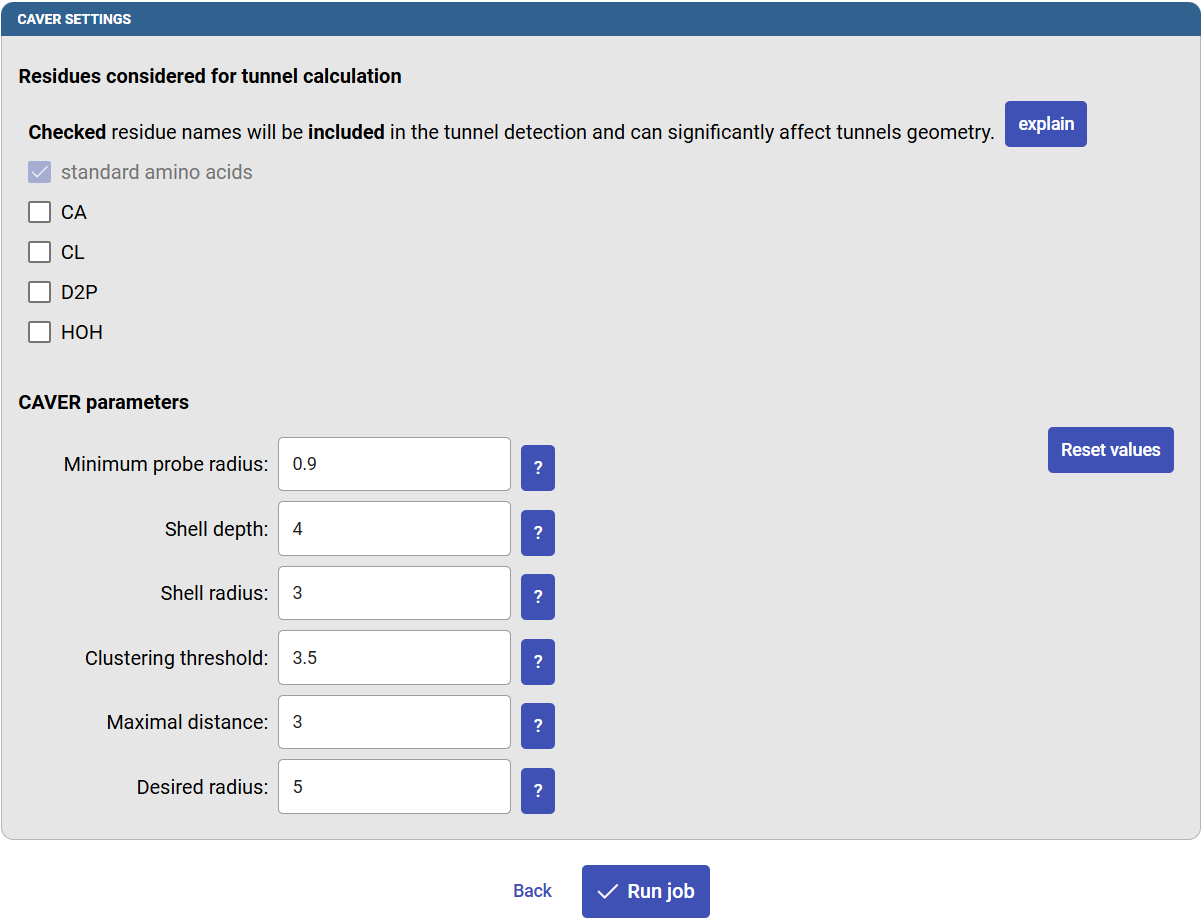


### Analyzing the tunnels

You will have an overview of the all the tunnels found and their statistics in the window “Tunnels information”. If no tunnels were found, you could try using a smaller probe size (Minimum probe radius) on the previous page. The Tunnel information panel shows the general properties of the tunnels, namely the bottleneck radius, the length of the tunnel, curvature and throughput. The checkboxes at the left-end of each tunnel allow you to display/undisplay the tunnel in the viewer down below.


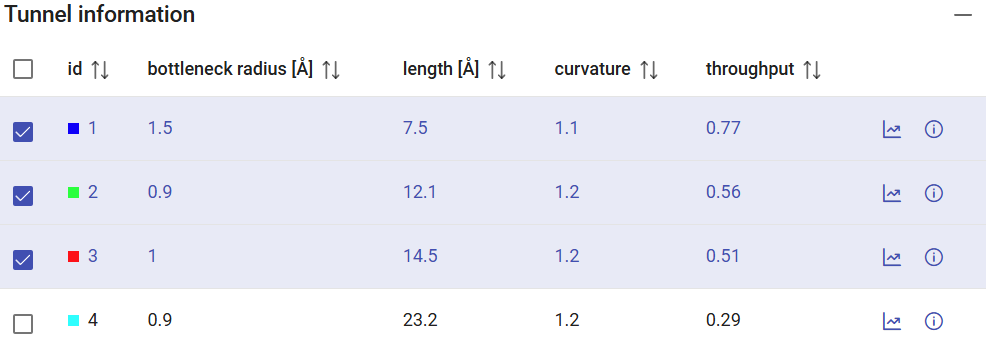


The small icons at the right-end of each tunnel let you take a look at several tunnel details. The “Show profile” icon (
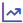
) displays the tunnel profile (the tunnel radius versus the length), which is very useful to identify the geometry and bottlenecks along the tunnel. You can superimpose profiles for the different tunnels, for comparison.


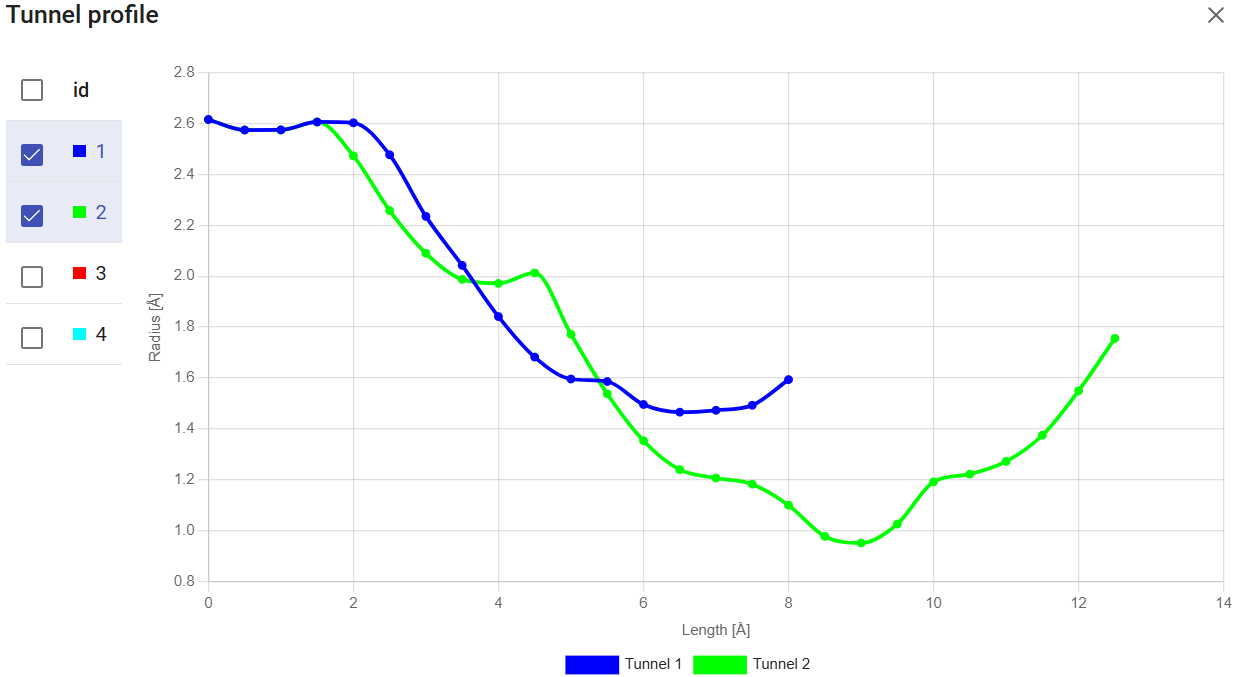


The “Show tunnel details” icon (
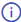
) will display all the details about the tunnel, like the bottleneck location and residues, the centerline, and the tunnel-lining residues and atoms.


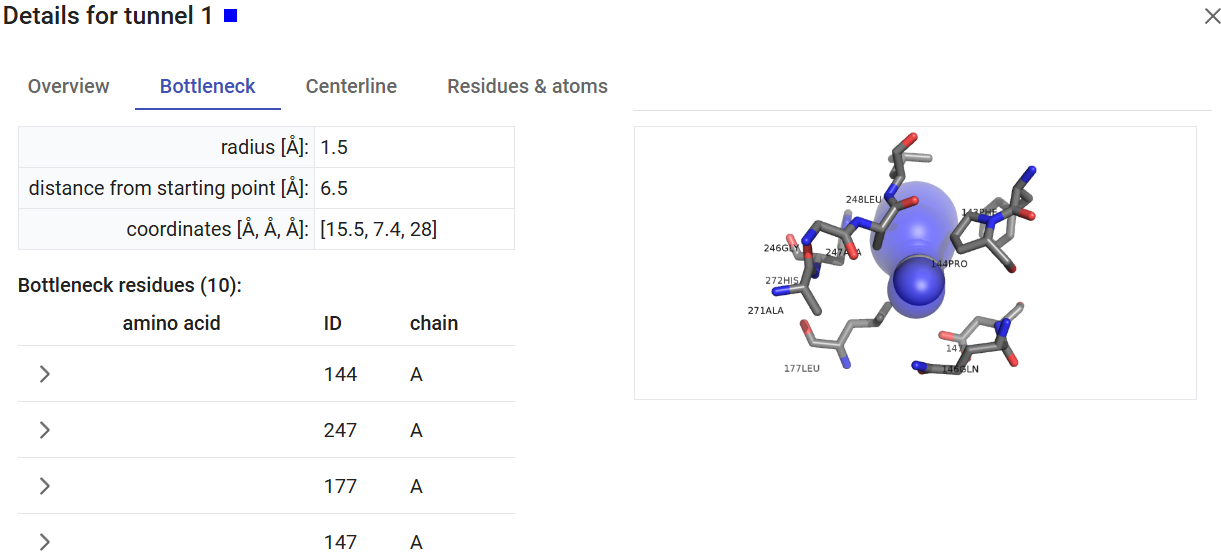


The viewer settings allow different visualization styles of the tunnels, either as opaque or transparent spheres. You can also select to visualize or not the pocket and the starting point for the tunnel calculation. You can export images directly from the Caver Web interface. For that, just click on the “screenshot” button (
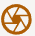
) on the viewer, adjust the cropped area, resolution, transparency, and download the image as the file in png format.


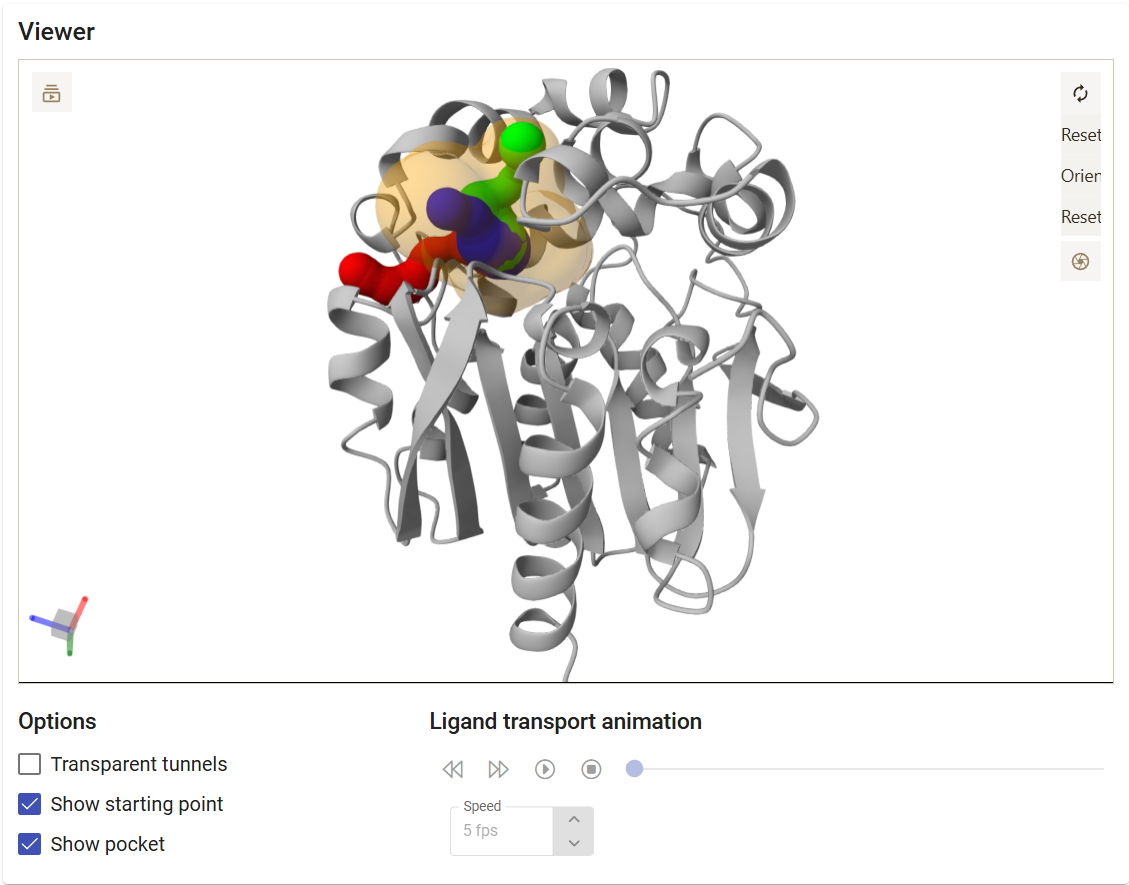


On the right-upper corner of the page you find a panel with the job details, such as the job-ID, the descriptive title, starting structure, date and time.

Below that, you can find several buttons, allowing you to download the visualization into a PyMOL session, the complete CAVER data folder as a zip file, the pockets calculated with the software fpocket, the CAVER configuration file, CAVER summary file, and several intermediate log files.

6
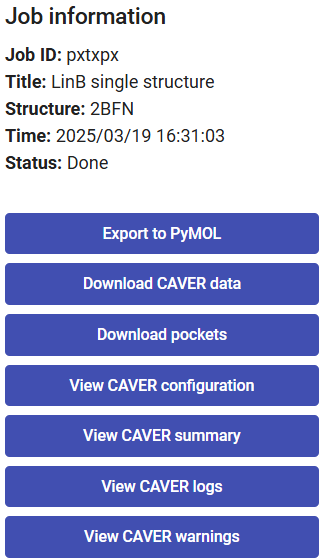


When you click on “Export to PyMOL”, select which objects you want to download, such as the pocket and tunnels, and click on “Export”. A zip file will be downloaded, then extract it to a regular folder and load the **pymol.pml** file into PyMOL. All the elements selected for download will be loaded in PyMOL and represented in a nice visualization that can be saved as a session for detailed inspection or even exported as an image for publication.


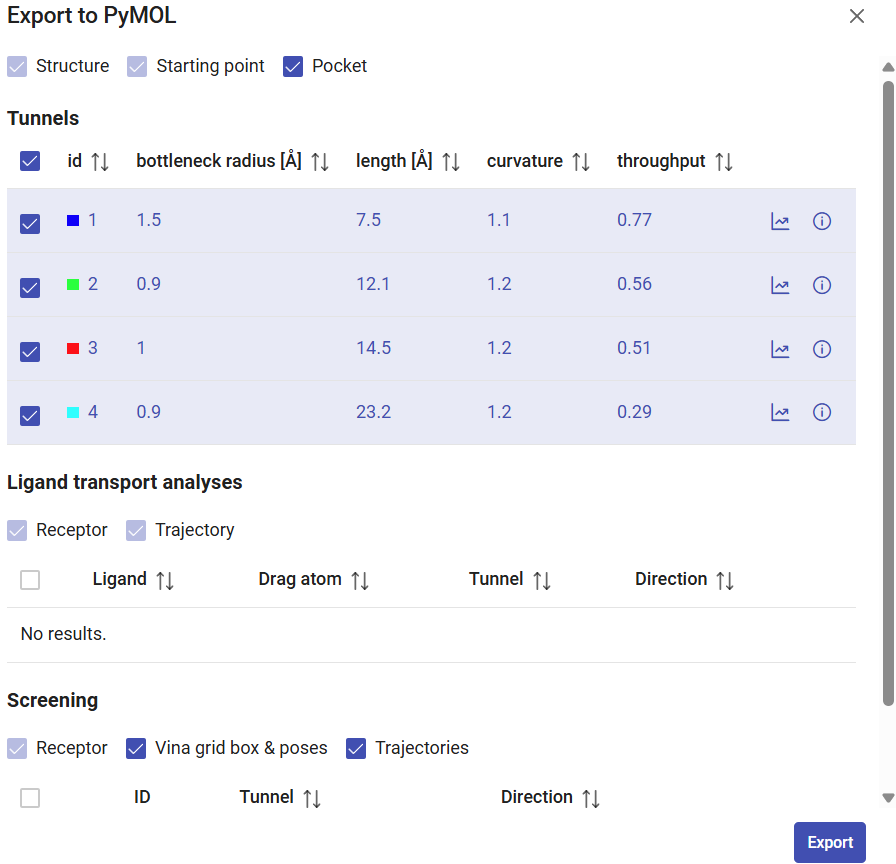


### Collecting the data

From the results above, tunnel 1 (blue) showed the widest bottleneck, the shortest length, and the lowest curvature. These factors are combined to compute the throughput of the tunnel, which was the highest among all the tunnels, and the reason why this tunnel was ranked first. The throughput is a quantification, based on the tunnel geometry, to tell us which tunnel is the easiest pathway between the active site and the surface. Therefore, tunnel 1 is the most likely to be used for actual transportation or molecules.

The previous steps can be repeated for other haloalkane dehalogenase enzymes, using their respective PDB IDs: DmmA (3U1T), DhaA (4HZG), DhlA (2YXP), and DbjA (3A2M). The main tunnel ranked as the 1^st^ one showed, for each of these enzymes, the properties listed in the table below:

| **Haloalkane dehalogenase** | **Bottleneck radius (Å)** | **Length (Å)** | **Curvature** | **Throughput** |
| --- | --- | --- | --- | --- |
| LinB | 1.5 | 7.5 | 1.1 | 0.77 |
| DmmA | 2.5 | 5.8 | 1.1 | 0.92 |
| DhaA | 1.6 | 9.2 | 1.2 | 0.74 |
| DhlA | 0.8 | 13.2 | 1.2 | 0.48 |
| DbjA | 2.3 | 2.8 | 1.1 | 0.92 |

The length of the tunnels varies from 2.8 Å to 13.2 Å. This is due to using the pocket center as the calculation starting point. DbjA and DmmA, with the shortest tunnels, have large, open and easily accessible catalytic sites, which makes their pockets span to the surface, thus bringing the center of the pockets close to the surface and shortening the length of the tunnels. Depending on the biological question addressed by the calculation, one can remake the analysis with a starting point defined by the catalytic residues and obtain different results for the tunnel length.

The haloalkane dehalogenases possess a broad substrate specificity and some of them show activity towards at least one of the poorly degradable toxic pollutants such as 1,2-dichloroethane, 1,2-dichloropropane or 1,2,3-trichloropropane (2). The enzymes with the narrower tunnels prefer smaller substrates and convert them with the higher catalytic efficiency. The preferred cognate substrate for DhlA with the narrowest tunnel is 1,2-dichloroethane, a small and hydrophobic compound. The preferred substrate for LinB is 1,2-dibromoethane, again a small and hydrophobic compound. DmmA with the widest tunnel prefers a longer molecule 4-bromobutanenitrile. Interestingly, also the enzymes with the narrowest tunnels, LinB and DhlA, can accommodate bulkier substrates thanks to conformational changes during the ligand binding. These conformational states can be sampled and studied with molecular dynamics simulations (MDs), which is the newest implementation in Caver Web 2.0, and will be described in the Use Case 2.

## **2. Binding and unbinding of ligands though the access tunnels of a haloalkane dehalogenase**

Load pre-calculated job for static structure: job [pxtxpx](https://loschmidt.chemi.muni.cz/caverweb/job/pxtxpx/)

For some enzymes with buried active sites, the binding of the substrate is the rate-limiting step, whereas for some others it is the release of the product. For situations like this, it can be very important to investigate the thermodynamics and trajectory of those molecules through the access tunnels of those enzymes. Such knowledge may help, for example, identifying the energy barriers and the location of the bottlenecks to the ligand transport, and thus focus the mutagenesis efforts to improve the catalytic properties of those enzymes. Yet in other cases, it may be relevant to investigate which tunnel, among the ones available, is more likely to transport a certain substrate or product. CaverDock (4) was designed to tackle this problem in a simple and inexpensive way, performing molecular docking of the ligands along a specific tunnel.

In this section, we will study the binding of substrate 1-chlorohexane, and the unbinding of the respective product 1-hexanol through the tunnels of haloalkane dehalogenase LinB. We will identify which tunnel is more likely to transport the substrate.

The usage of Caver Web to study the transport of ligands through access tunnels consists of several sequential stages:

- Selecting the system and calculating the tunnels (previous section)
- Selecting the ligand
- Selecting the tunnels
- Setting up advanced parameters
- Analyzing the results

### Selecting the ligand

The CaverDock calculations are performed under the “Ligand transport with CaverDock” panel.

There are several means to setup the ligands to be studied with CaverDock. To input the ligands, you can either fetch them by name from the PubChem database, upload your own files in any format supported by OpenBabel (e.g., mol, mol2, sdf, pdb or pdbqt), paste a ligand SMILES code or ZINC accession code, or draw the structure of the molecule in the ligand editor.

The SMILES for substrate [1-chlorohexane](https://pubchem.ncbi.nlm.nih.gov/compound/1-Chlorohexane) is CCCCCCCl. Click on the “Paste” pane, paste this code, and click on “Process”. You could add a list of multiple ligands with different codes, one per line, to run multiple CaverDock calculations in a single submission. You can rename every ligand to its chemical name or any acronym of your choice. Under the field “Name”, change the name from CCCCCCCl to “chlorohexane”.


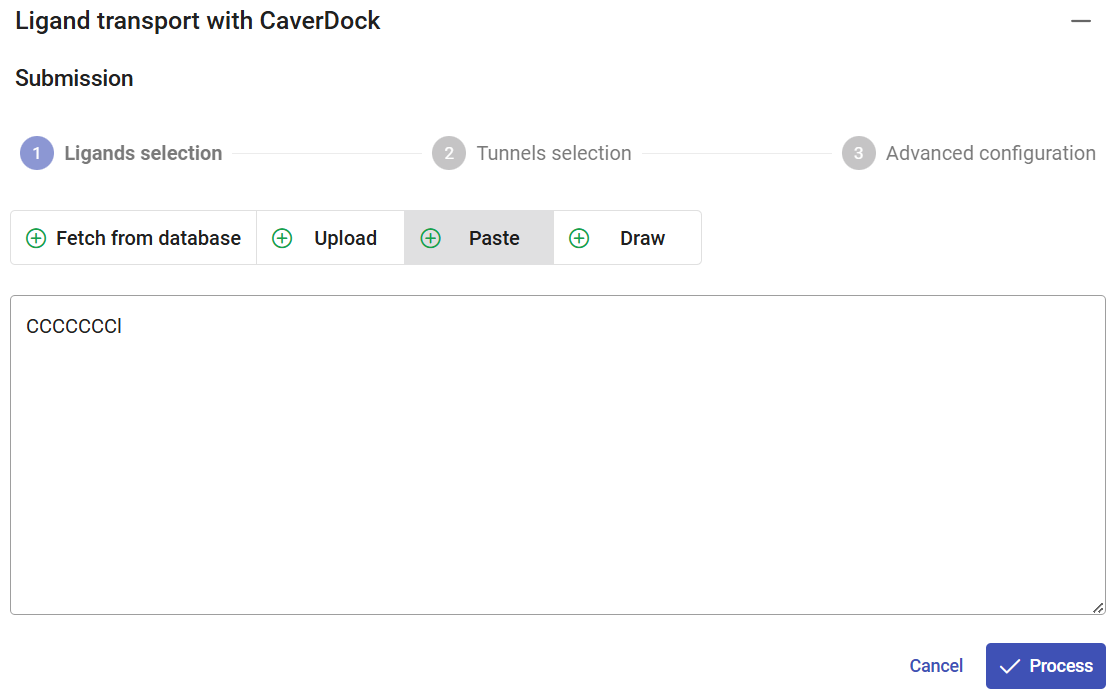


You can also add another ligand to the list for a single submission. For the sake of this example, click on the “Draw” pane, and draw the 2D formula of [1-hexanol](https://pubchem.ncbi.nlm.nih.gov/compound/1-Hexanol) as in the figure below, and click on “Process”. Under the field “Name”, change the name from CCCCCCO to “hexanol”.


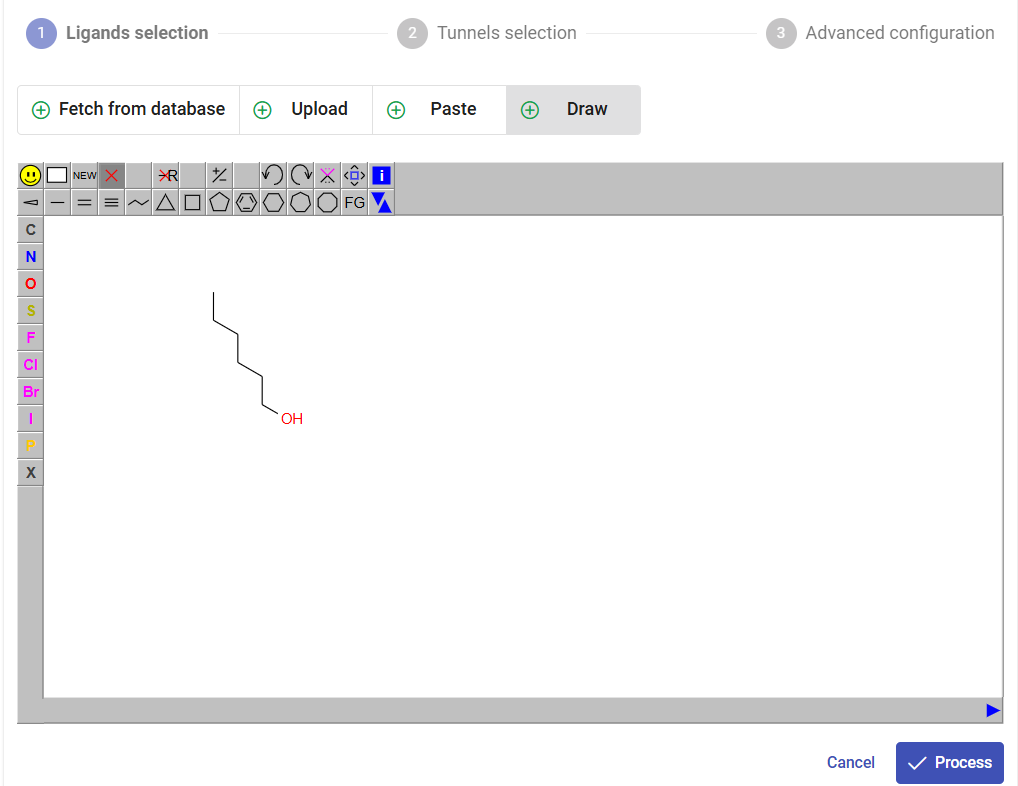


Now you have a list of two ligands to be analyzed by CaverDock. Since chlorohexane is a substrate, which travels through the tunnel from the protein surface to the active, select the direction “in”. Since hexanol is a product travelling through the tunnel from the active site to the surface, select the direction “out”. Click on “Next”.


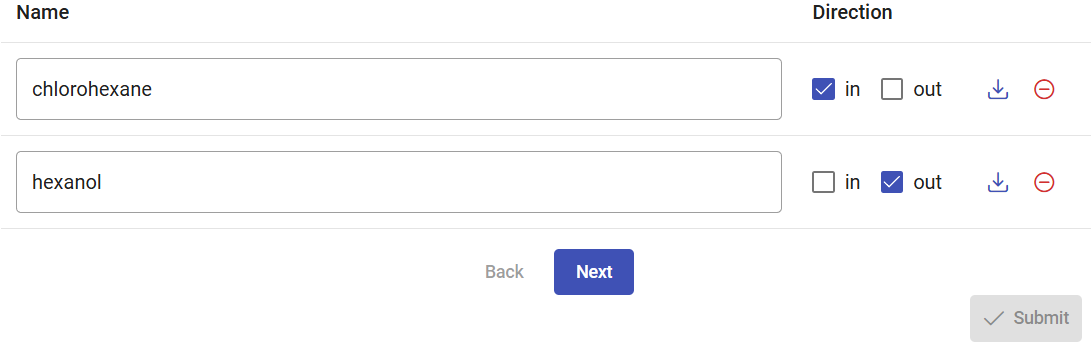


### Selecting the tunnels

Among the tunnels obtained for LinB (previous section), since tunnels 1 and 2 are superimposed for a good part of their topology (see the image below), we will investigate tunnels 1 and 3 to perform a comparative study.


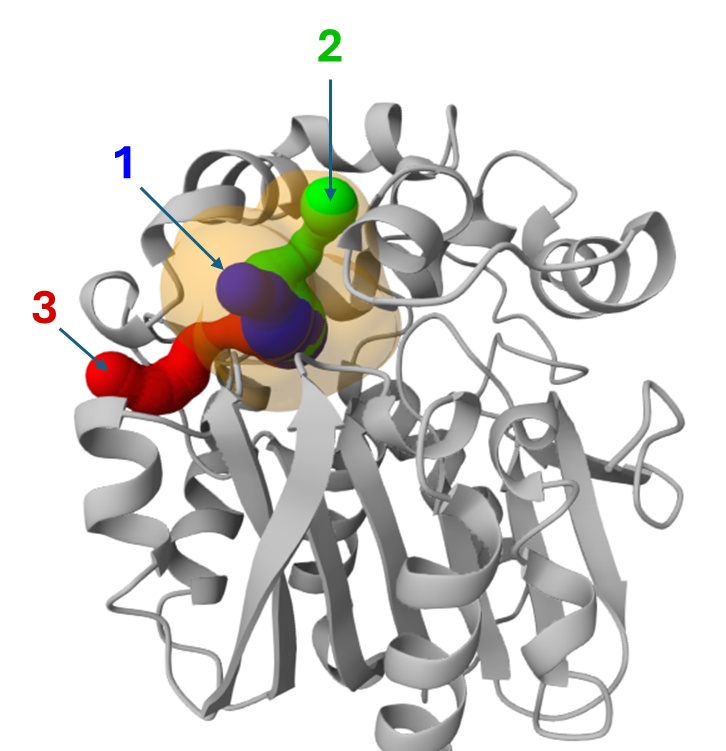


Click on “Next” for advanced configurations, or on “Submit” to proceed immediately with the CaverDock calculations.


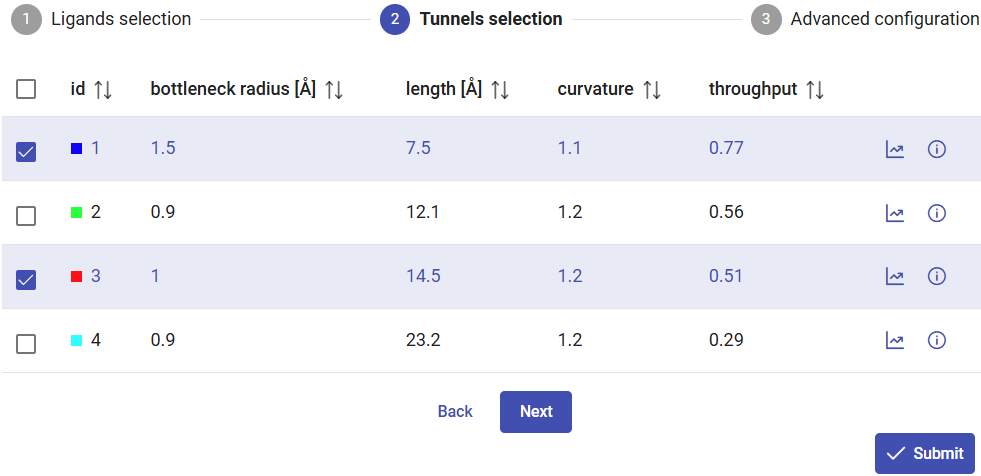


### Setting up advanced parameters

Under the “Advanced configuration” pane, you can change the discretization step of the tunnel to refine the docking step along the tunnel.

In addition to the “lower-bound” trajectory, you can also choose to compute the “upper-bound” trajectory, which will calculate a continuous trajectory of the ligand, where the ligand is not allowed to flip while moving through the tunnel. Keep in mind that if you select this option, it will take a significantly longer time to compute due to the heuristics and backtracking required for the continuity of the trajectory, and the resulting energy can be excessively and artificially high. Currently, we recommend users to consider mainly the lower-bound trajectory and profile, and to use upper-bound trajectories for educational purposes only. In this tutorial we will only calculate the “lower-bound” trajectory.


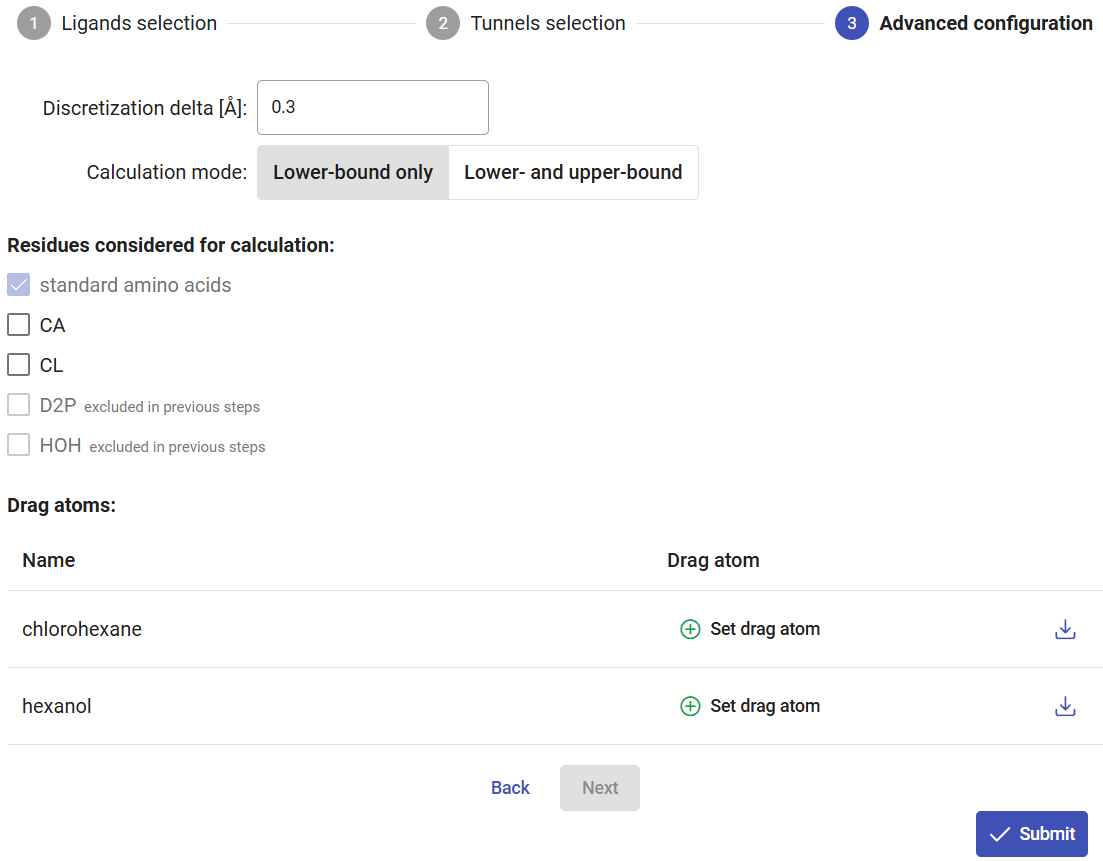


If you click on the rightmost button available for each ligand, you can view and download the pdbqt file generated for that ligand. You can also specify an atom to be “dragged” along the tunnel, instead of the default center of mass of the ligand. This feature may be useful when you want to specify the reacting atom to be directed towards the active site. Alternatively, whenever your ligand is large and you already have an idea how it may bind in the active site, you can also guide the binding process by specifying an atom that you know to be more buried in the binding cavity, and use it as a dragged atom. In our case, since the chlorine atom is connected to the reacting carbon atoms, and therefore it is a good proxy for guiding the substrate towards the active site, and you can specify it as a ‘drag atom’. For that, click on the “Set drag atom” button. For 1-chlorohexane, select the chlorine atom (CL7_7) and click on “Confirm”. For hexanol, this feature is less relevant, but we can select the hydroxyl to be dragged out towards the solvent. For that, select the oxygen atom (O7_7), and then click on “Confirm”.


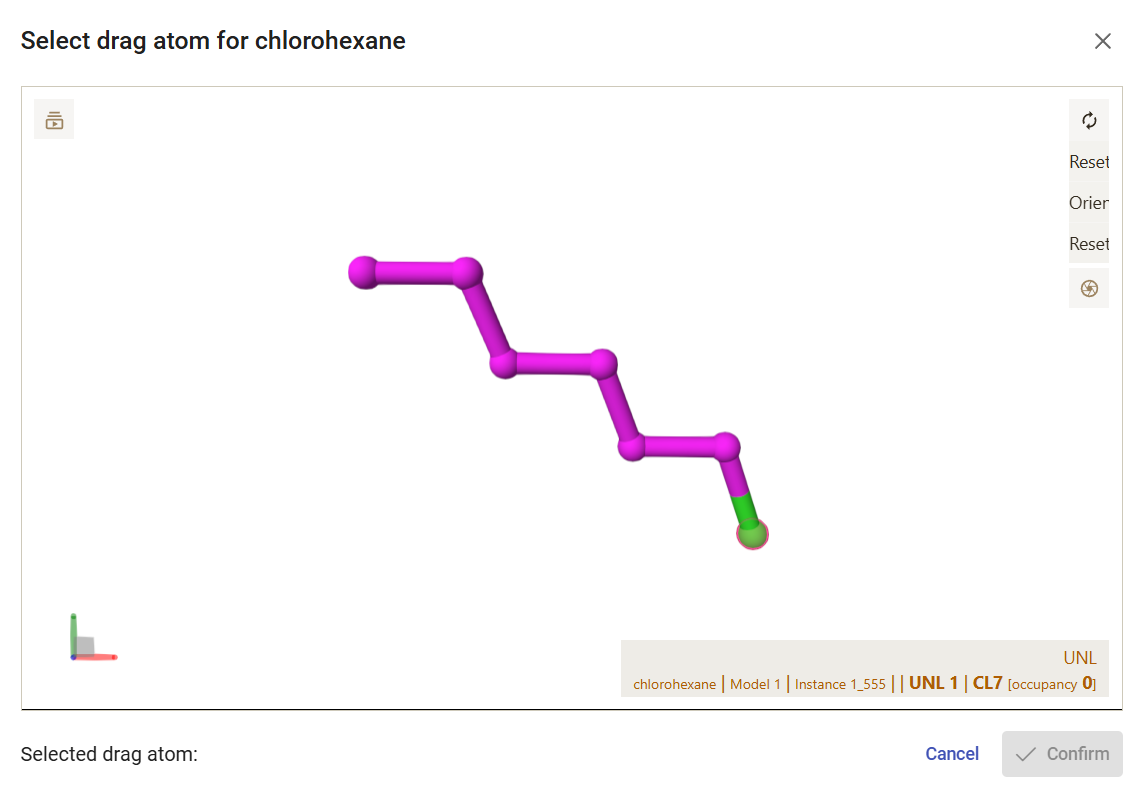


Finally, click on “Submit” to run all the CaverDock calculations. The calculations will take between several minutes to a few hours.

### Analyzing the results

After the CaverDock calculations are finished, you will find them in the Results panel with a green check mark on the left. Each calculation lists the name of the ligand that was submitted, with the ID of the dragged atom, the tunnel that was used, and the direction (“in” or “out”). On the right you can find several buttons to visualize and download the results, which are described below.


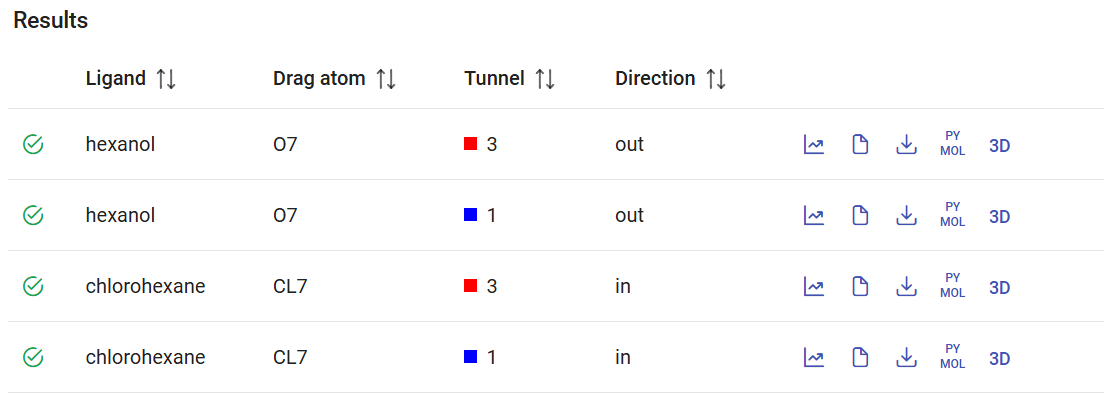


If you click on “Show results” (
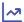
 ), it will display the CaverDock energy profiles, which shows the change in the binding energy of the ligand moving along the tunnel. If the user-defined direction was “in”, the “surface” will be at the left of the graph (Distance 0) and the “active site” at the right. The opposite will happen for the “out” direction. Click on “show results” for the calculation with chlorohexane in tunnel 1 and the “in” direction. The energy at the surface (at the left) we call E_surface_. Soon after the surface, the energy increased to its maximum value during the trajectory, which we call E_max_. At the left-most part of the plot we have the energies at the active site, with energies that we call E_bound_. If you hover your mouse over each point, you will see the details for that point. If you click one point, you can add one of the tags: E_bound_, E_max_, or E_surface_. The respective energies will appear listed on the upper-right corner, together with two new values: the activation energy E_a_ (E_a_ = E_max_ – E_surface_ for the “in” direction, and E_a_ = E_max_ – E_bound_), and the energy difference between the bound state and the surface ΔE_BS_ (ΔE_BS_ = E_bound_ – E_surface_). These values provide important inform about the kinetics (the lower the E_a_, the faster the process) and thermodynamics (the more negative the ΔE_BS_, the more prone is the ligand bind the active site instead of the surface) of the ligand binding process. In practical terms, we recommend specifying the E_surface_ as the minimum-energy near the surface, because often there is a shallow valley at the tunnel mouth where the ligand binds with non-zero energy, or the energy-plateau if there is one. E_max_ should be specified at the maximum energy point along the tunnel. E_bound_ is the minimum-energy value near the binding site, because sometimes the energy can spike when the ligand is forced too close to the bottom of the cavity.

We want to highlight that CaverDock is not a replacement for detailed (un)binding MD simulations or free-energy methods. As an approximate docking-based approach, it is well suited for rapid screening or rough estimation. Although its accuracy improves significantly when multiple protein snapshots are considered (5), the resulting energy profiles remain approximate and are best used for comparative purposes rather than absolute energetic interpretation.

If you click on the left arrow in the bottom, you will obtain the CaverDock profile for the next calculation, which is chlorohexane travelling inwards through tunnel 3. After we assign the E_bound_, E_max_, or E_surface_ points, we find that the activation energy E_a_ (the energy barrier to the transport) is much higher than it was for tunnel 1 (3.5 vs. 0.9 kcal/mol). Interestingly, the energy at the surface was also lower for tunnel 1 than for tunnel 3, suggesting that chlorohexane is more attracted to the mouth of tunnel 1 than tunnel 3.

For the release of hexanol from the active site from tunnels 1 and 3, we found E_a_ of 1.2 and 5.8 kcal/mol, respectively. None of these barriers are extremely high, which suggests that both tunnels could potentially be used for the transport of small ligands, which is in agreement with the literature (6). Altogether, these results suggest that tunnel 1 is the preferred pathway for the transport of both the substrate and product in and out of the active site, respectively.


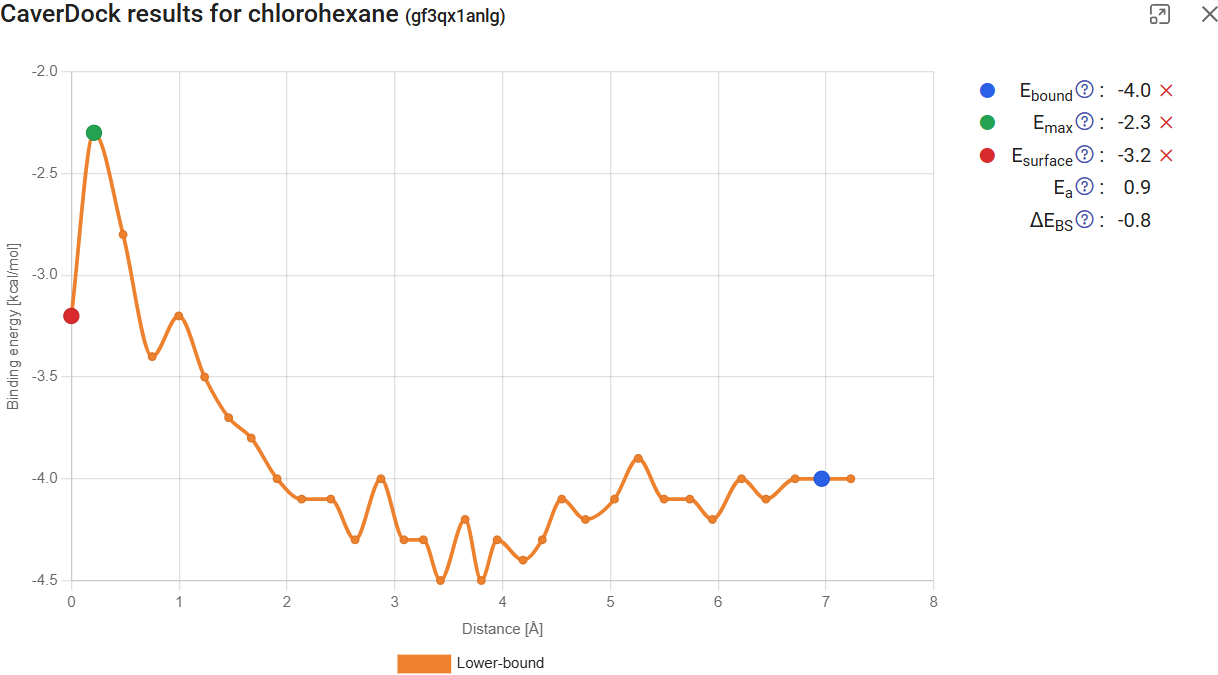


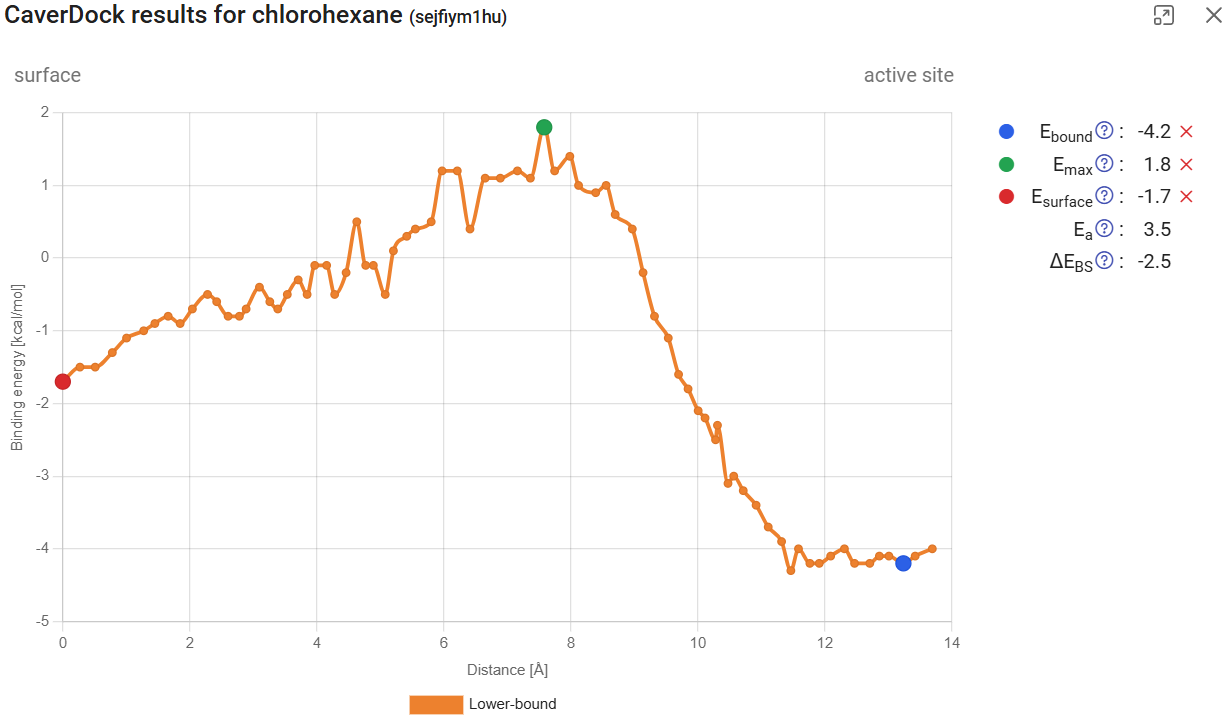


The left-most button, “Show ligand in viewer” (
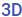
), allows you to visualize the corresponding tunnel and ligand trajectory in the viewer up above. You can play the trajectory as a movie, jump to a specific step of the CaverDock calculation, or even save a specific snapshot as an image with the “Screenshot” button in the viewer (
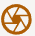
). For example, click on the
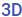
 button for the trajectory of chlorohexane within tunnel 3.


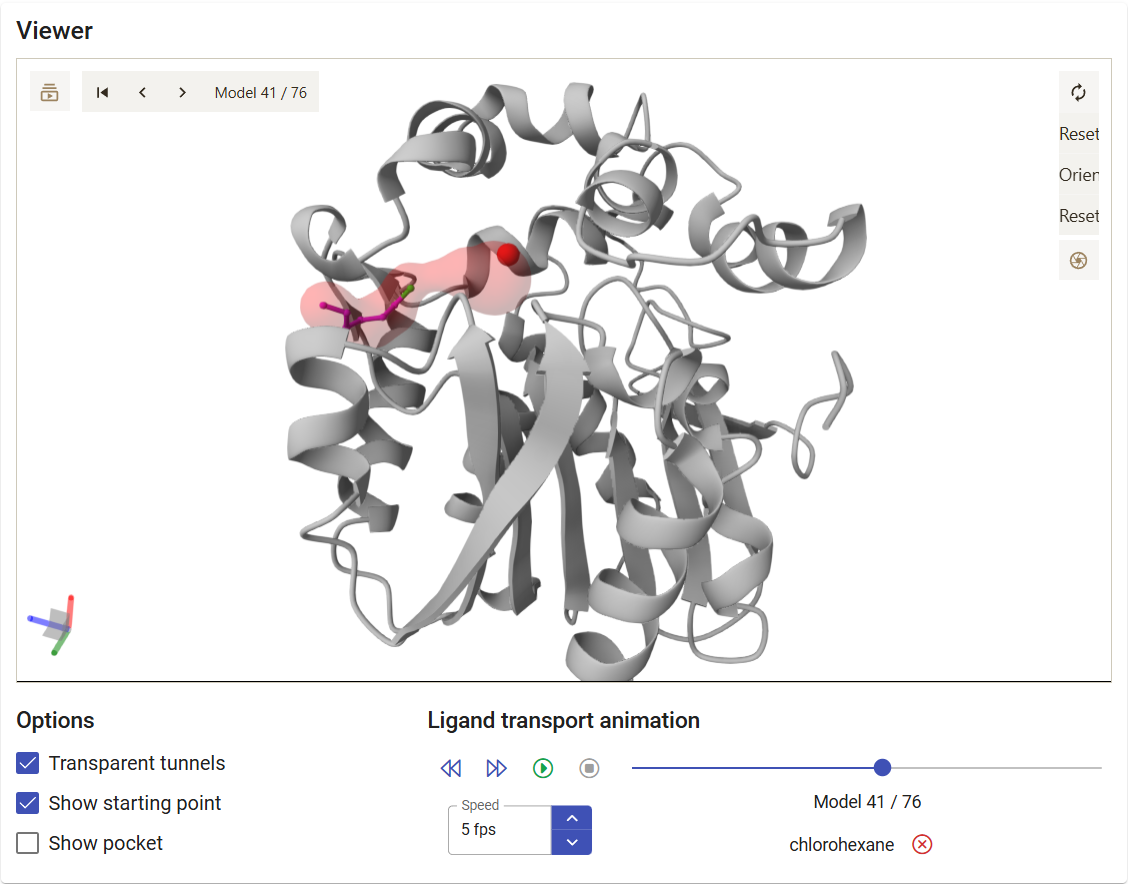


If you click on the “Download PyMOL scrip” button (
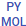
), a zip file is downloaded. Extract it to a regular folder, and load the **pymol.pml** file to PyMOL. You will visualize your protein, with the tunnels and the ligand. If you click on the “play” button in PyMOL, the ligand will move according to the trajectory predicted with CaverDock.

The “Log file” button (
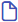
) allows you to obtain details on the CaverDock calculation, and the “download raw data” allows (
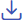
) to download the CaverDock calculation folder as a zip file.

## **3. Virtual screening of substrates in the active site and tunnels of a haloalkane dehalogenase**

Load pre-calculated job for static structure: job [pxtxpx](https://loschmidt.chemi.muni.cz/caverweb/job/pxtxpx/)

This section allows the user to screen multiple of molecules on the binding site and tunnels of the proteins. This can be very useful for the virtual screening of drugs, to identify the metabolites that can bind a specific receptor, or to find potential new substrates or inhibitors for some enzyme. For this task, we enabled the possibility to screen all the FDA/EMA-approved drugs in a single submission, and the possibility to enter a user-personalized list of ligands of interest.

This section of Caver Web consists on the following steps:

- Selecting the system and calculating the tunnels (previous sections)
- Selecting the ligands
- Selecting the grid box
- Selecting the tunnels
- Analyzing the data

### Selecting the ligands

After calculating the tunnels in your protein of interest (follow the steps in the previous sections), the virtual screening is available at the “Screening” panel. To define which ligands you want to screen, you can select the option “Use all FDA/EMA-approved drugs”. This can be particularly useful if you are searching for repurposing approved drugs towards some specific disease and target of your choice.

You can also specify ligands by uploading their files (e.g., mol, mol2, sdf, pdb or pdbqt format), which can be done by browsing or “drag and drop” multiple files from your computer. You can also enter a list of ZINC codes (one per line), or enter a list of SMILES codes.

For this example, select the option “Enter SMILES codes”, copy and paste the list of SMILES codes below, which describe several halogenated compounds (as obtained from [PubChem](https://pubchem.ncbi.nlm.nih.gov/compound/1-Hexanol)) that are potential substrates for the haloalkane dehalogenases, and then click on “Next”.

| **SMILES code** | **Compound name** |
| --- | --- |
| C(C(CBr)Br)Br | 1,2,3-Tribromopropane |
| C(C(CCl)Cl)Cl | 1,2,3-Trichloropropane |
| C(CBr)Br | 1,2-Dibromoethane |
| CC(CBr)Br | 1,2-Dibromopropane |
| C(CBr)CBr | 1,3-Dibromopropane |
| C(CI)CI | 1,3-Diiodopropane |
| C(CBr)Cl | 1-Bromo-2-chloroethane |
| C(CCl)CBr | 1-Bromo-3-chloropropane |
| CCCCBr | 1-Bromobutane |
| CCCCCCBr | 1-Bromohexane |
| C(CCl)OCCCl | 1-Chloro-2-(2-chloroethoxy)ethane |
| CCCCCl | 1-Chlorobutane |
| CCCCCCCl | 1-Chlorohexane |
| CCCCI | 1-Iodobutane |
| CCCCCCI | 1-Iodohexane |
| CCCI | 1-Iodopropane |
| C=C(CCl)Cl | 2,3-Dichloroprop-1-ene |
| CCC(C)I | 2-Iodobutane |
| C(CC#N)CBr | 4-Bromobutanenitrile |
| C1CCC(C1)Cl | Chlorocyclopentane |


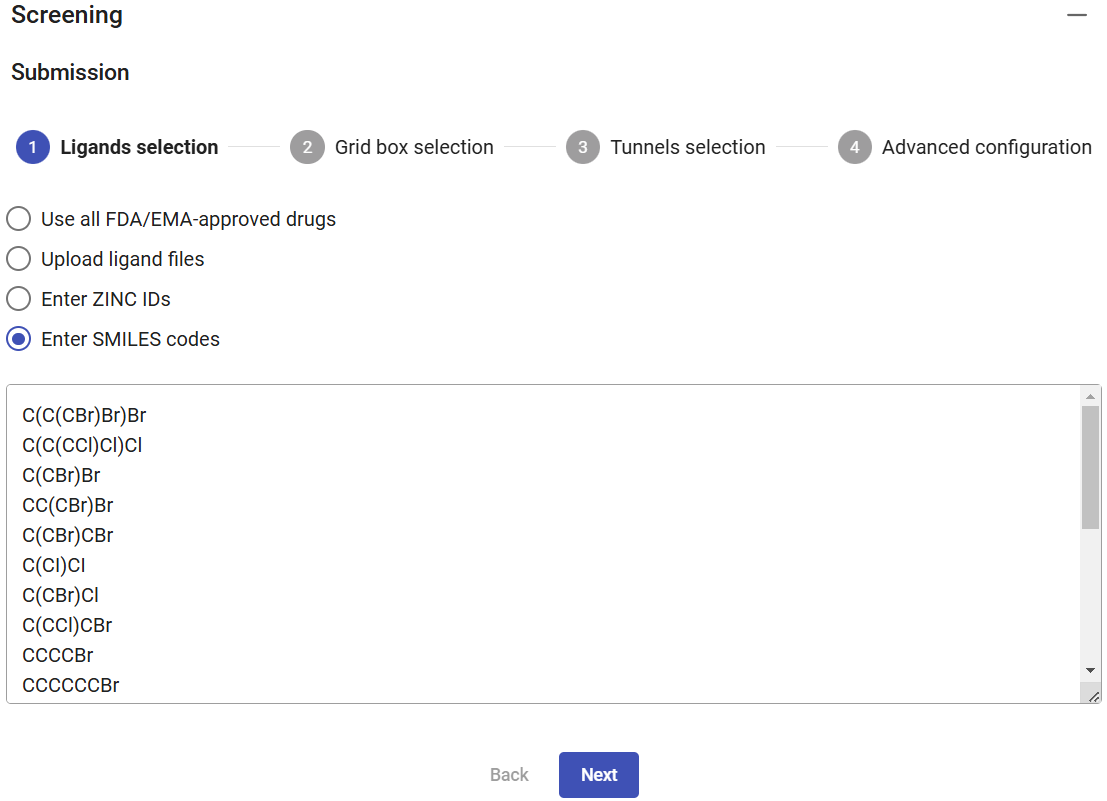


### Selecting the grid box

Now you have to select the center and size of the grid box to perform the docking. Two presets are available: the “Starting point” option centers the box at the origin point that was used for calculating the tunnels and defines a symmetric cubic box with 20 Å in each side; the “Pocket” option centers the box in the center of the pocket originally selected and defines the size as 3 Å beyond the pocket limits. You can always readjust the center and dimensions of the grid box manually.

For this example, select the “Starting point” and keep the default dimensions. Click on “Next”.


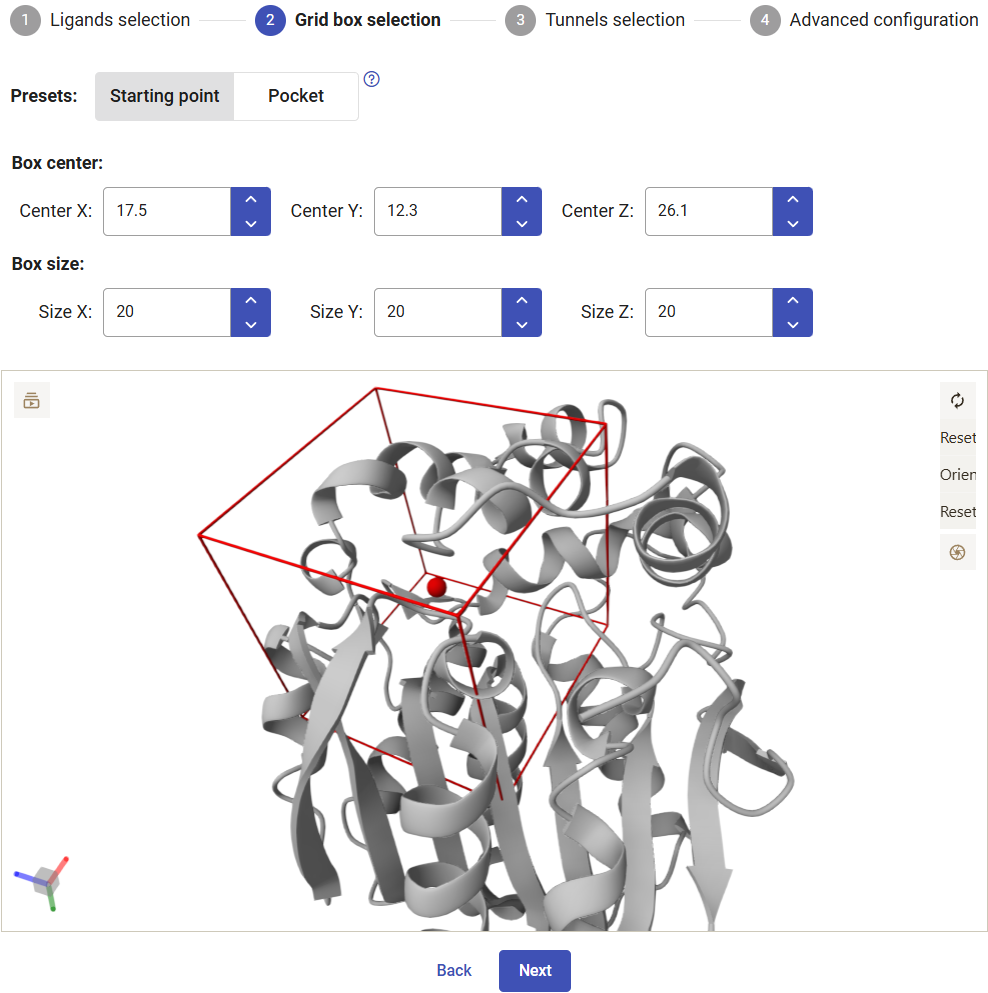


### Selecting the tunnels

For running the CaverDock calculations with each one of the ligands, you have to specify a tunnel. Here we will use tunnel 1, which showed the best abilities to transport our substrate chlorohexane (previous section). Click on “Submit”. The calculations will take several hours to complete.


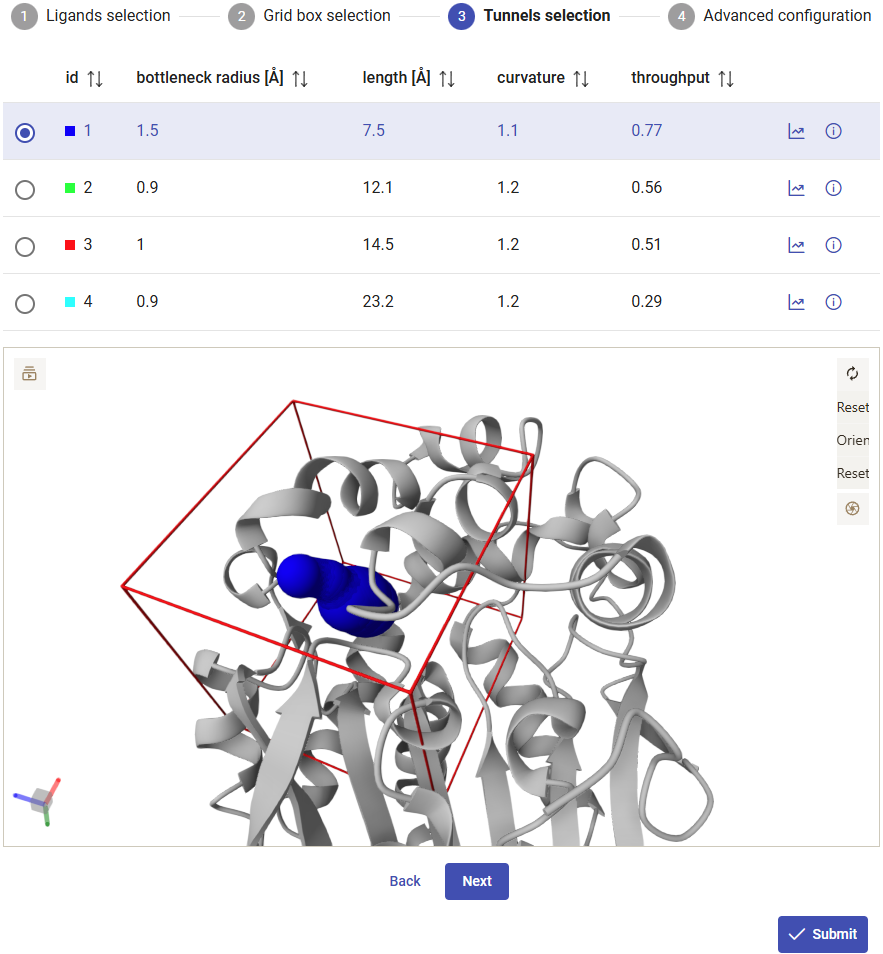


### Analyzing the data

After the screening jobs have finished, they can be found in the Results panel (which lists all the screening jobs) with a green check mark on the left if they finished successfully. Each job has an ID, the selected tunnel for the CaverDock calculation, and the respective direction (“in” is the only direction currently allowed).

At the right-end of each job you can find several icons: “Show results” (
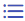
) to analyze all the details in an interactive window, “Download raw data in zip archive” (
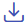
) to save the original calculation folder, and “Download PyMOL script” (
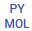
).


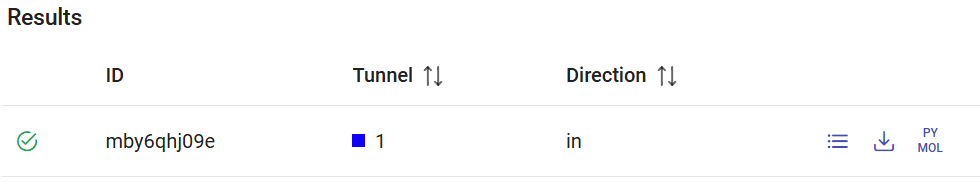


The screening job mby6qhj09e corresponds to the previous calculation with the list of substrates. Click on the respective “Show results” button. You will see a window displaying the list of ligands with best binding energies in the binding site. For each compound, you can see the binding energy at the active site (from docking with AutoDock Vina), and parameters from the CaverDock profile: the maximum energy and the energy difference between the bound state and the surface (ΔE_BS_). On the right-most side, you find icons that allow you to visualize the CaverDock profiles (
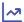
 ) and download the raw data (
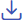
) for each compound individually.

You can also sort the list of compounds by any of these energy values by clicking on the arrows next to the respective labels. In this case, sort by ascending energy in the active site, and select the three-top compounds with the most negative energies. Click on “Compare energies of selected ligands”.


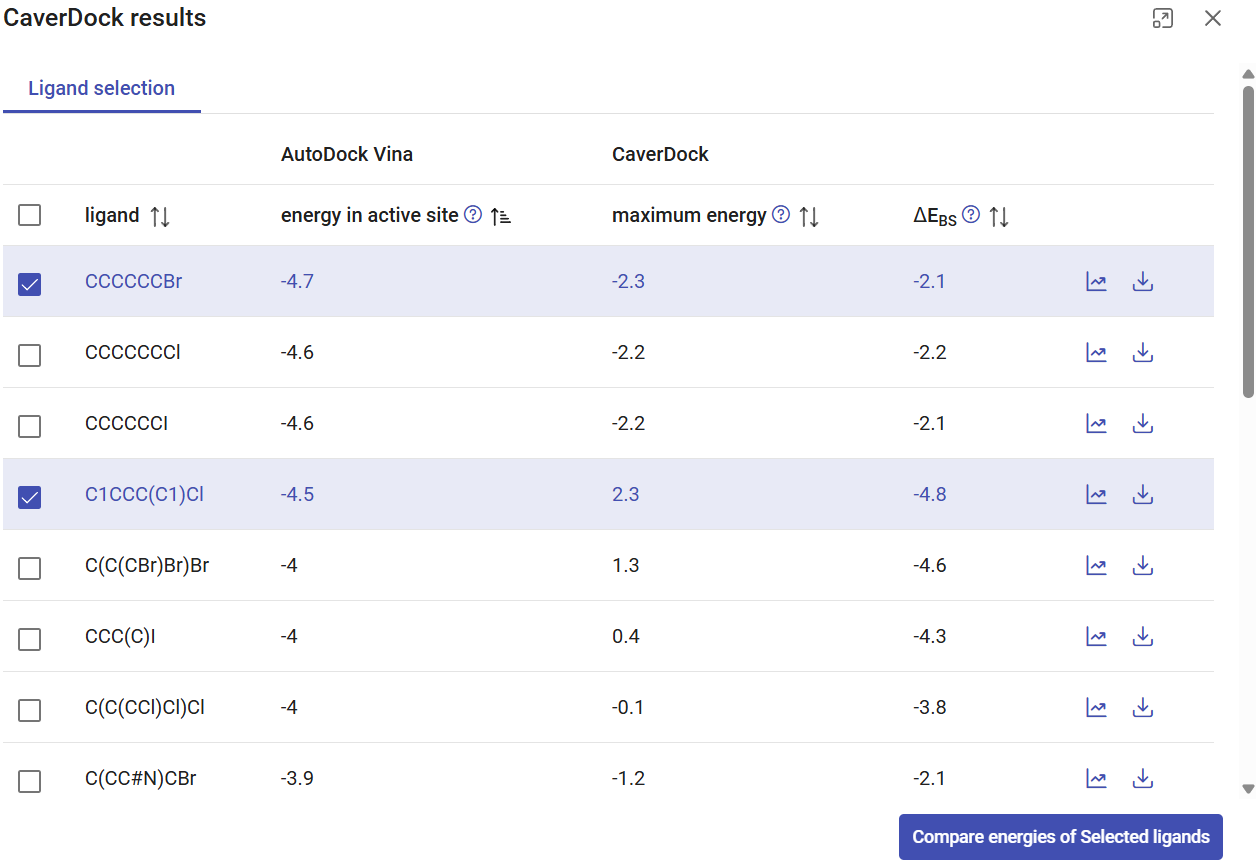


A new tab will open, with the CaverDock profiles superimposed for the three selected compounds. You can see that substrate CCCCCCBr (1-bromohexane) showed the best affinity in the active site (-4.7 kcal/mol). C1CCC(C1)Cl (chlorocyclopentane) showed the most favorable ΔE_BS_ difference (-4.8 kcal/mol) but a higher energy barrier (E_a_ 2.5 kcal/mol), which suggests hindrance on its transport due to its bulkiness. The fact that all these compounds have negative binding energies in the active site (with affinities ranging between -4.7 to -2.9 kcal/mol) and relatively low E_a_ suggests that most of them can have favorable thermodynamics for binding haloalkane dehalogenase LinB, and hence they may be potential substrates for this enzyme. We already know this to be true, from the literature (2). Experimentally validated LinB favorite substrate, 1,2-dibromoethane (C(CBr)Br) is among them with the active site affinity -2.9, E_a_ 0.0, ΔE_BS_ -2.9 kcal/mol. The fact that it did not show the absolute best values among all the substrates suggests that, although the access of this substrate through the tunnels is favorable, it is not the main determinant for the catalytic conversion of this substrate. In the window, you can have multiple tabs with the individual CaverDock profiles and with the comparison of selected ligands.


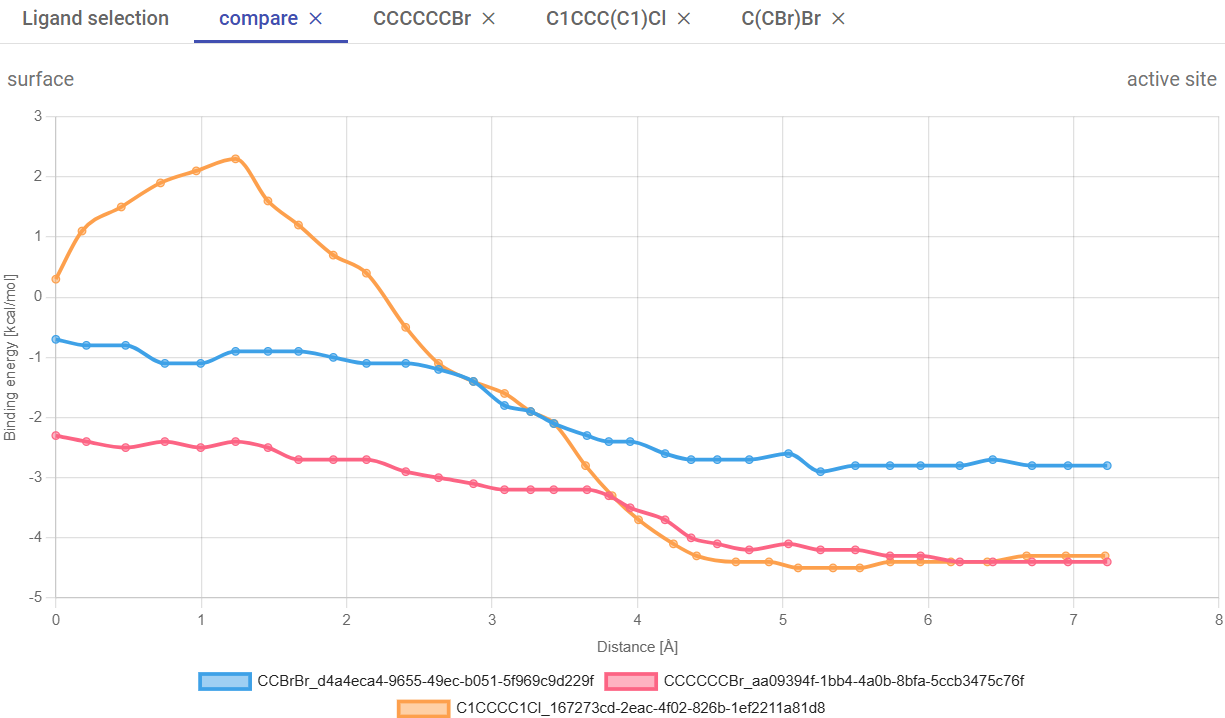


Now, close the results window. For job *hc80b8bnoh*, click on “Download PyMOL script” (
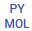
), save the zip file, extract it to a regular folder, and load the **pymol.pml** file into PyMOL. You will visualize many objects, namely the protein structure (the original structure), all the tunnels, pocket, and the results from the screening. For clarity, you can undisplay all the objects, leaving only tunnel 1 (“*tun_cl_1*”).

Under “*screening*”, you can find the docking and CaverDock results. Now display the object “*receptor*” to represent the protein. If you display the objects under “*vina*”, you will see the grid box (used in the docking) and the docking “*poses*” for the different ligands, which you can analyze individually in detail and investigate all the existing interactions and contacts. By default, you will see the best-ranked binding modes, but if you click on the “next” or “previous” buttons you can visualize alternative poses from Autodock Vina (ordered by the energy scores, from best to worse).


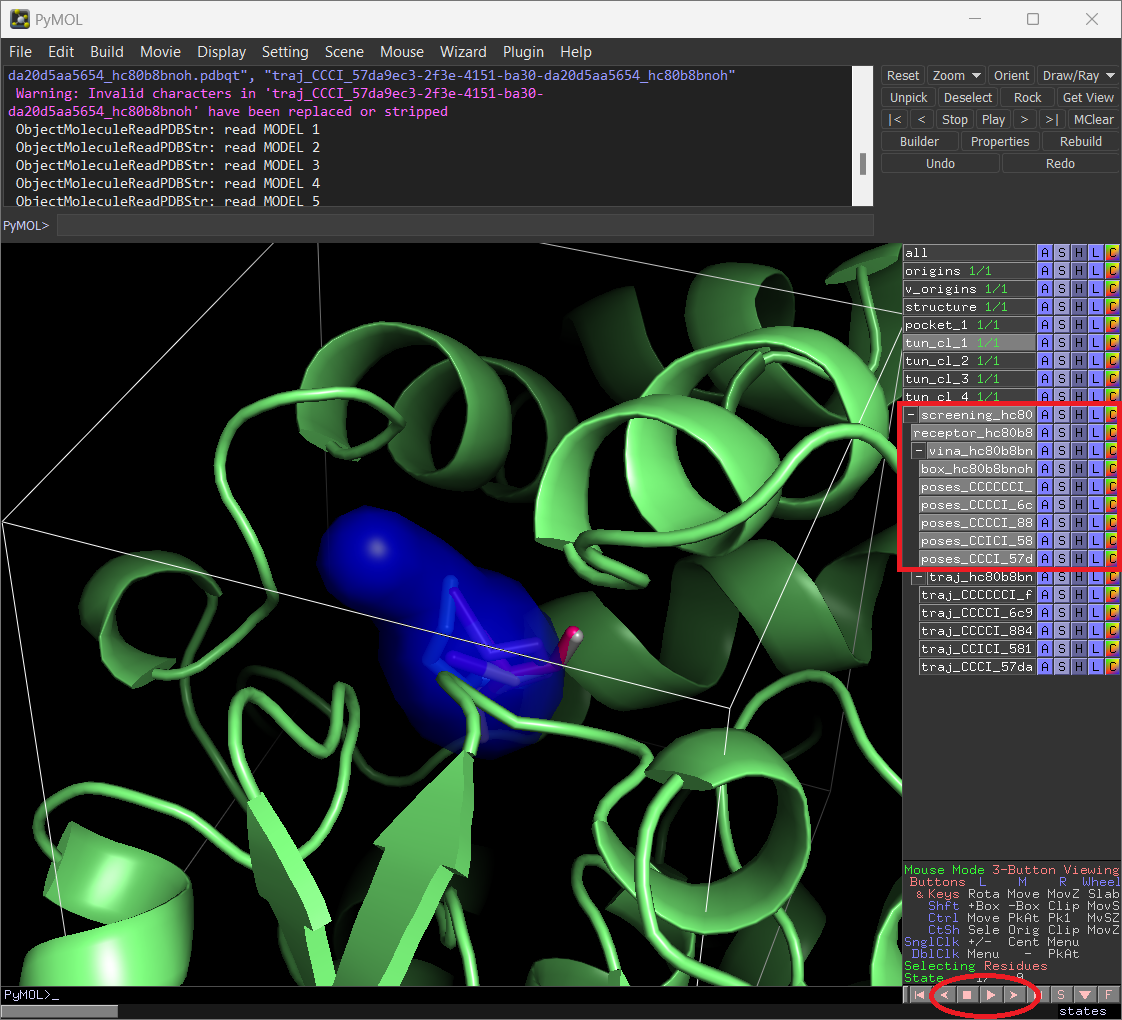


Instead, if you display the “*receptor*” and the “*traj*” objects, you will visualize the protein and the CaverDock poses for the different compounds. If you click on the “play” button in PyMOL, you will visualize the complete trajectories of those ligands, which you can analyze in more detail.


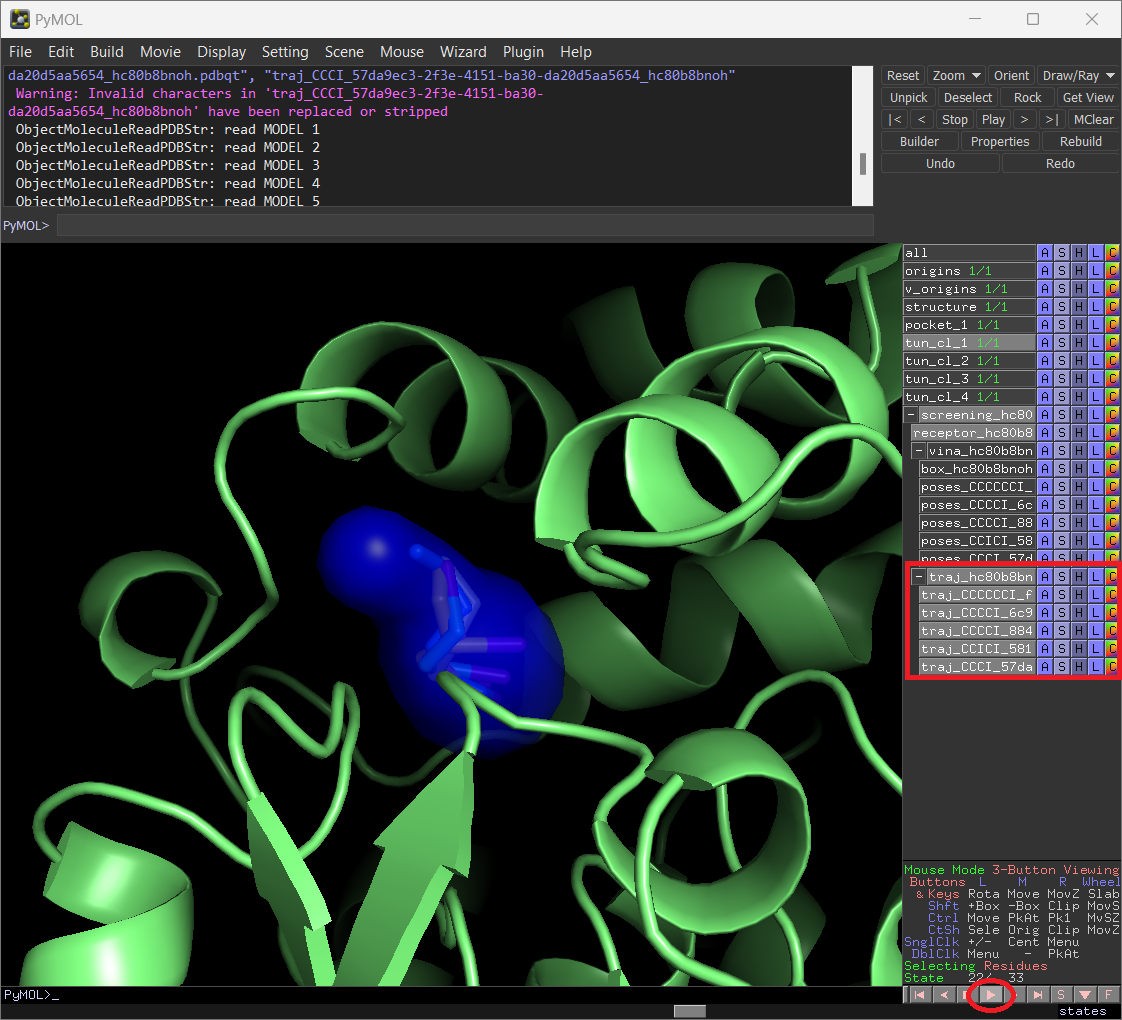


## References

1. Marques,S.M., Dunajova,Z., Prokop,Z., Chaloupkova,R., Brezovsky,J. and Damborsky,J. (2017) Catalytic Cycle of Haloalkane Dehalogenases Toward Unnatural Substrates Explored by Computational Modeling. *J. Chem. Inf. Model.*, **57**, 1970–1989.

https://doi.org/10.1021/acs.jcim.7b00070

2. Koudelakova,T., Chovancova,E., Brezovsky,J., Monincova,M., Fortova,A., Jarkovsky,J. and Damborsky,J. (2011) Substrate specificity of haloalkane dehalogenases. *Biochemical Journal*, **435**, 345–354.

https://doi.org/10.1042/BJ20101405

3. Chovancová,E., Pavelka,A., Benes,P., Strnad,O., Brezovsky,J., Kozlikova,B., Gora,A., Sustr,V., Klvana,M., Medek,P., *et al.* (2012) CAVER 3.0: a tool for the analysis of transport pathways in dynamic protein structures. *PLoS Comput. Biol.*, **8**, e1002708.

https://doi.org/10.1371/journal.pcbi.1002708

4. Vavra,O., Filipovic,J., Plhak,J., Bednar,D., Marques,S.M., Brezovsky,J., Stourac,J., Matyska,L. and Damborsky,J. (2019) CaverDock: a molecular docking-based tool to analyse ligand transport through protein tunnels and channels. *Bioinformatics*, **35**, 4986–4993.

https://doi.org/10.1093/bioinformatics/btz386

5. Vavra,O., Tyzack,J., Haddadi,F., Stourac,J., Damborsky,J., Mazurenko,S., Thornton,J.M. and Bednar,D. (2024) Large-scale annotation of biochemically relevant pockets and tunnels in cognate enzyme–ligand complexes. *Journal of Cheminformatics*, **16**, 114.

https://doi.org/10.1186/s13321-024-00907-z

6. Brezovsky,J., Babkova,P., Degtjarik,O., Fortova,A., Gora,A., Iermak,I., Rezacova,P., Dvorak,P., Smatanova,I.K., Prokop,Z., *et al.* (2016) Engineering a de Novo Transport Tunnel. *ACS Catal.*, **6**, 7597–7610.

https://doi.org/10.1021/acscatal.6b02081
