# Supplementary material for: Caver Web 2.0: analysis of tunnels and ligand transport in dynamic ensembles of proteins
Source: Nucleic Acids Res. 2025 May 8;53(W1):W132–42. doi: 10.1093/nar/gkaf399 (PMC12230698; doi:10.1093/nar/gkaf399)
Supplement: gkaf399_Supplemental_Files [file gkaf399_supplemental_files.zip › UseCase2_dynamic_mode_R1.docx]

**Use Case 2: Study of enzyme tunnels and ligand transport in a dynamic ensemble**

**List of Content**

[1. Studying the dynamic properties of access tunnels in a haloalkane dehalogenase 2](#_Toc194167581)

[Selecting the protein structure, setting the tunnel starting point and the CAVER parameters 2](#_Toc194167582)

[Running MD simulation and calculating the tunnels 2](#_Toc194167583)

[Analyzing the MD simulation 3](#_Toc194167584)

[Analyzing the dynamic tunnels 4](#_Toc194167585)

[2. Studying the binding of ligands though dynamic tunnels of a haloalkane dehalogenase 8](#_Toc194167586)

[Selecting the ligand 8](#_Toc194167587)

[Selecting the tunnels 9](#_Toc194167588)

[Setting up advanced parameters 10](#_Toc194167589)

[Analyzing the results 11](#_Toc194167590)

[References 15](#_Toc194167591)

## **1. Studying the dynamic properties of access tunnels in a haloalkane dehalogenase**

Load pre-calculated job for molecular dynamics: job [xbgsjm](https://loschmidt.chemi.muni.cz/caverweb/job/xbgsjm/)

The access tunnels of enzymes with buried active sites change their topology and geometric properties with the dynamical changes intrinsic to all macromolecules. Some tunnels may be permanently open, but others may open and close in time (e.g., in some snapshots their bottleneck radius decreases below a certain threshold) (1–3). In this section, we will run a 5 ns molecular dynamics (MD) simulation on haloalkane dehalogenase LinB, using YASARA (4, 5), and then study the variation of the tunnel properties during this simulation using CAVER 3.03 (6) implemented in Caver Web 2.0.

The usage of Caver Web in molecular dynamics mode consists of several sequential stages:

- Selecting the protein structure, setting the tunnel starting point and the CAVER parameters
- Running MD simulation and calculating the tunnels
- Analyzing the MD simulation
- Analyzing the dynamics tunnels

### Selecting the protein structure, setting the tunnel starting point and the CAVER parameters

Follow the same first steps as described in Use Case 1 (for single structures):

1. Enter the PDB code “2BFN” and click on “Download file”
2. Click on “Next” to use the default monomeric structure
3. Define the starting point for the tunnel calculation by selecting the first catalytic pocket with pocket ID #1 and click on “Next”
4. In the next page, you can optionally enter a job description title and your e-mail address.

### Running MD simulation and calculating the tunnels

Under the “Molecular dynamic setting” panel, select the option “Enable molecular dynamics”, and new options will appear. You can select which residues and molecules will be present in the MD simulation. You may decide to include co-factors, if they are intrinsic part of your protein, and crystallographic water molecules. But we recommend excluding crystallographic ligands, which may induce the native tunnels to become larger than in the native proteins. In this case, click on HOH to include the water molecules in the MD simulation.

You can adjust the “YASARA parameters” that define some of the conditions in which the MD will be carried out. For example, you can modify the pH, temperature, MD duration, and the frequency to save the snapshots (which will affect the final number of snapshots that will be saved and analyzed). In this case, specify the temperature as 298.15 K (25.0 °C), a duration of 5000 ps (5 ns) and the save interval as 100 ps, so you will obtain 50 snapshots for the analysis.


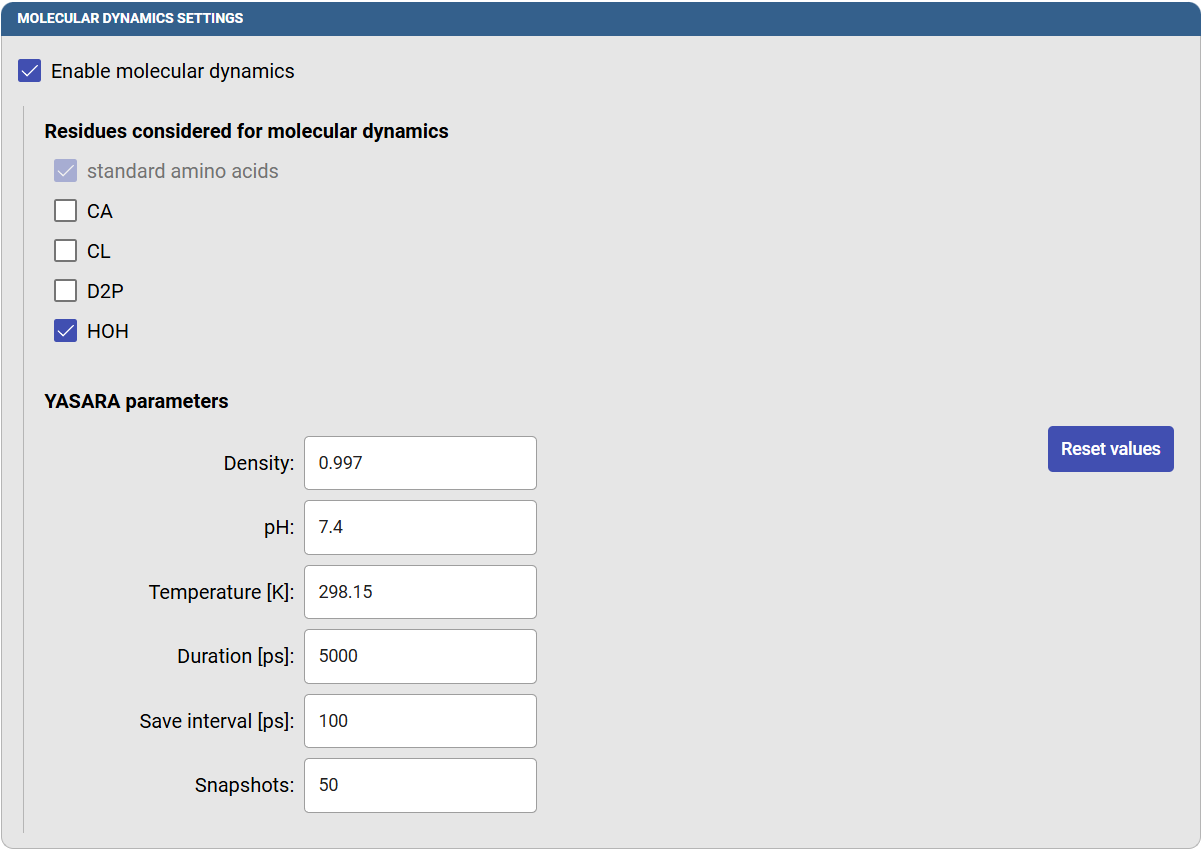


In the next panel, you can define the CAVER settings for the tunnel calculation, as described in Use Case 1 (for single structures). For this tutorial, use the default settings and click on “Run job” at the bottom. Depending on the size of your protein and the duration of the MD simulation that you defined, the calculation may take several hours to complete.

### Analyzing the MD simulation

On the right-upper corner of the page, you have a panel with the job details, and several buttons to download files. Some of these have been described in Use Case 1.

At the bottom, you can download or visualize the YASARA report (zip or html files, respectively) on the MD trajectory that was performed. The YASARA report includes many useful details on the MD simulation such as: details on the system, the variation in time of different energy components, surface area, hydrogen bonds, secondary structures, residue contacts, radius of gyration, root-mean square deviation (RMSD), root-mean square fluctuation (RMSF), among others.

If you click on “Snapshots (zip)”, you can download the saved snapshots from the MD in a zip file. These snapshots contain the system after removing the solvent and ions and pre-aligned by superimposing the Cα atoms of the protein.

When you click on “Export to PyMOL”, a zip file will be downloaded. Extract it to a regular folder and load the **pymol.pml** file into PyMOL. All the protein snapshots will be included and synchronized with the respective tunnels, which can be displayed sequentially if you click on the “play” button in PyMOL.


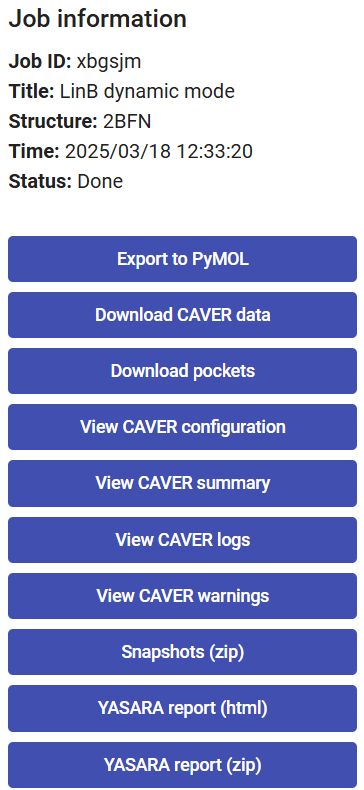


### Analyzing the dynamic tunnels

The tunnels calculated on the MD ensemble, using the CAVER settings defined on the previous page, will be listed in the panel “Tunnel information”. In this case, the information for each tunnel is: the total number of snapshots in which it was detected, the bottleneck radius, maximum bottleneck radius observed in the MD, tunnel length, curvature and throughput. Whenever applicable, the values are reported as an average value with the standard deviation. After the MD simulation, more tunnels were found than in the static structure of LinB (compare with Use Case 1). Moreover, the existing tunnels changed significantly their radius, length and curvature, and some of them were not even detected in most of the snapshots. We can see that the first two tunnels were detected very frequently ≥94% of the snapshots, while tunnel 3 was observed in only 29% of the snapshots. The checkboxes at the left-end of each tunnel allow you to display/undisplay the different tunnels in the viewer, down below. Select only the top 4 tunnels to visualize, since all the others appear scarcely in the MD (7% of the snapshots).


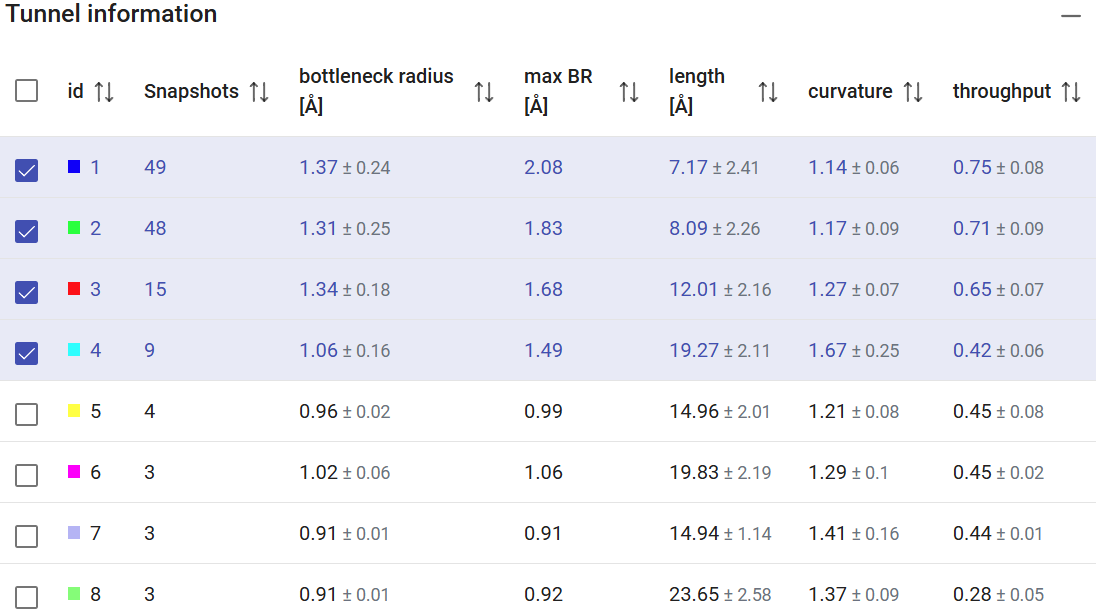


Below the “Tunnel information” is the “Bottleneck heatmap”. This map describes, for each tunnel, the bottleneck radius along the MD simulation by the color intensity. It allows easily assessing how frequently each tunnel was detected (according to the probe radius specified before), and how wide or narrow it was throughout the MD. If you hover your mouse on a particular tunnel and snapshot, you will see the bottleneck radius of the tunnel at that exact snapshot. In this case, we can easily identify that tunnel 1 was at its widest stage in snapshot 15, with a bottleneck radius of 2.08 Å. If you hover the mouse on the right end of the panel, hidden buttons appear and you can zoom the heatmap in and out and download it as image file in png format.


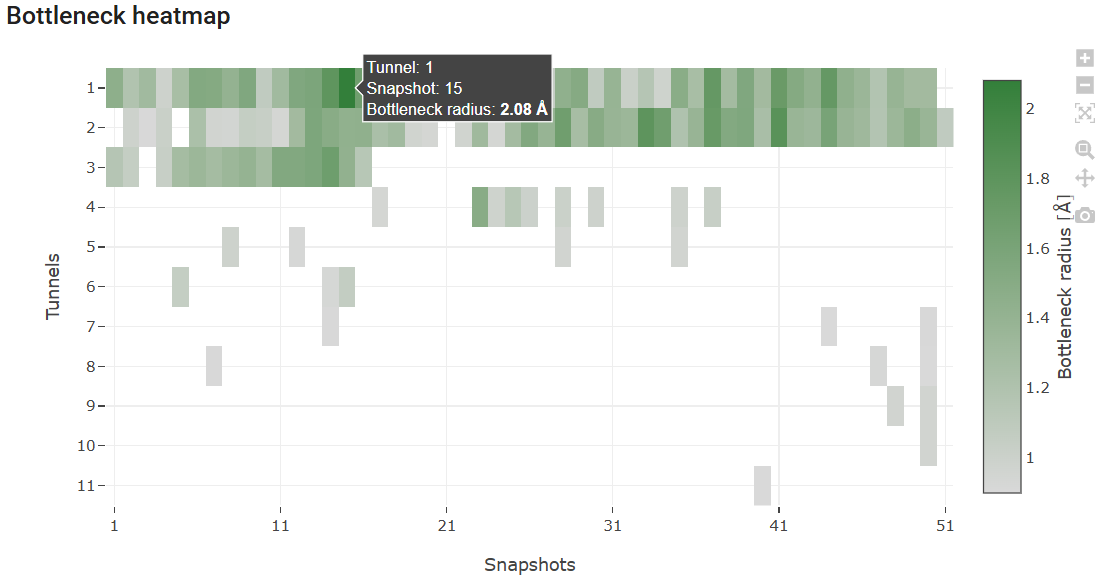


The viewer shows the motions of the protein backbone and the tunnels dynamics during the MD simulation. The tunnels selected in the panel above are displayed, and the user can analyze, for each snapshot, how their geometry and topology changed from snapshot to snapshot, and when they were detected. Under the viewer window are several buttons to stop/play the animation and move the snapshots back and forth, a slider that can be used to quickly move the MD back and forth to the desired snapshots, and a field to control the playback speed.


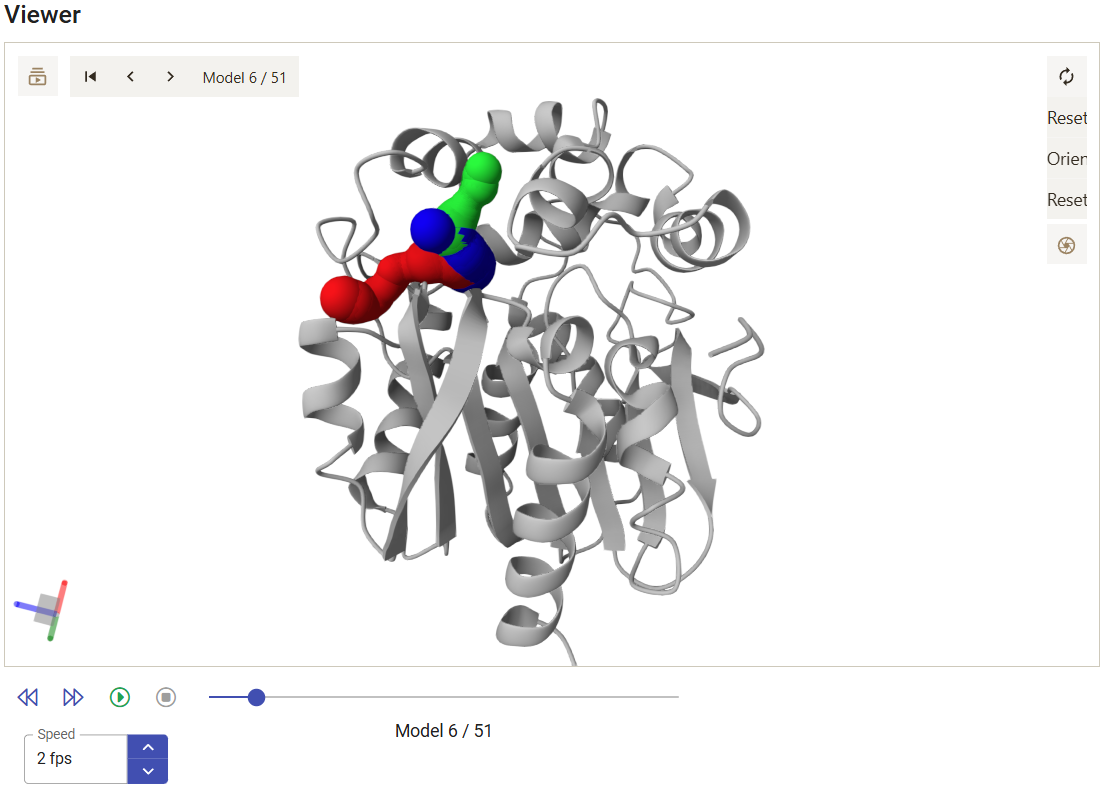


From the 3D Viewer one can see that the preference order and the topology of the three main tunnels are the same in the dynamic ensemble as in the corresponding crystal structure of LinB (see the Use Case 1). This may not always be the case (for example, tunnel 4 is completely different in the static structure and in the dynamic ensemble). We can see that those tunnels varied significantly in the ensemble. From the statistical information on the Tunnel information panel, we find that tunnel 1 reached a maximum bottleneck of 2.08 Å, which is wider than the bottleneck radius in the crystal structure, although the average was lower than in the static structure (1.37 ± 0.24 Å). On the other hand, tunnel 2 revealed more dramatic differences. While tunnel 2 was barely detected in the crystallographic structure (bottleneck radius 0.9 Å), in the dynamic ensemble the average bottleneck radius was substantially wider (1.31 ± 0.25 Å) with a maximum bottleneck radius of 1.83 Å, which is sufficient for the transport of small molecules. A similar situation was found with tunnel 3. The throughput values of the top-3 tunnels, which quantify the likelihood of the tunnels having biochemical function in the transport of ligands, are similar or higher in the dynamic ensemble compared to the crystal structure. Conversely, while in the crystal structure tunnel 4 was identified (in spite of the low throughput), in the ensemble it ranked #8, and it was detected in only in 3 snapshots (6%), which shows how rare and practically irrelevant this tunnel can be.

##

## **2. Studying the binding of ligands though dynamic tunnels of a haloalkane dehalogenase**

Load pre-calculated job for dynamic: job [xbgsjm](https://loschmidt.chemi.muni.cz/caverweb/job/xbgsjm/)

In this section, we will study the binding of substrate 1-chlorohexane, and the unbinding of product 1-hexanol through the dynamic tunnels of haloalkane dehalogenase LinB, as obtained after performing a 5 ns MD simulation. We will identify which tunnel is more likely to transport the substrate.

The usage of Caver Web to study the transport of ligands through dynamics tunnels consists of several sequential stages:

- Selecting the system and calculating the tunnels (previous section)
- Selecting the ligand
- Selecting the tunnels
- Setting up advanced parameters
- Analyzing the results

### Selecting the ligand

The CaverDock calculations are performed under the “Ligand transport with CaverDock” panel.

There are several means to setup the ligands to be studied with CaverDock. To input the ligands, you can either fetch them by name from the PubChem database, upload your own files in any format supported by OpenBabel (e.g., mol, mol2, sdf, pdb or pdbqt), paste a ligand SMILES code or ZINC accession code, or draw the structure of the molecule in the ligand editor.

In Use Case 1, section2, we described how to import or build a ligand. Please repeat those steps to add substrate [1-chlorohexane](https://pubchem.ncbi.nlm.nih.gov/compound/1-Chlorohexane) and [1-hexanol](https://pubchem.ncbi.nlm.nih.gov/compound/1-Hexanol) to be analyzed here. You can simply “Paste” the SMILES CCCCCCCl to specify chlorohexane, add other line with the SMILES CCCCCCO for hexanol, and click on “Process”.


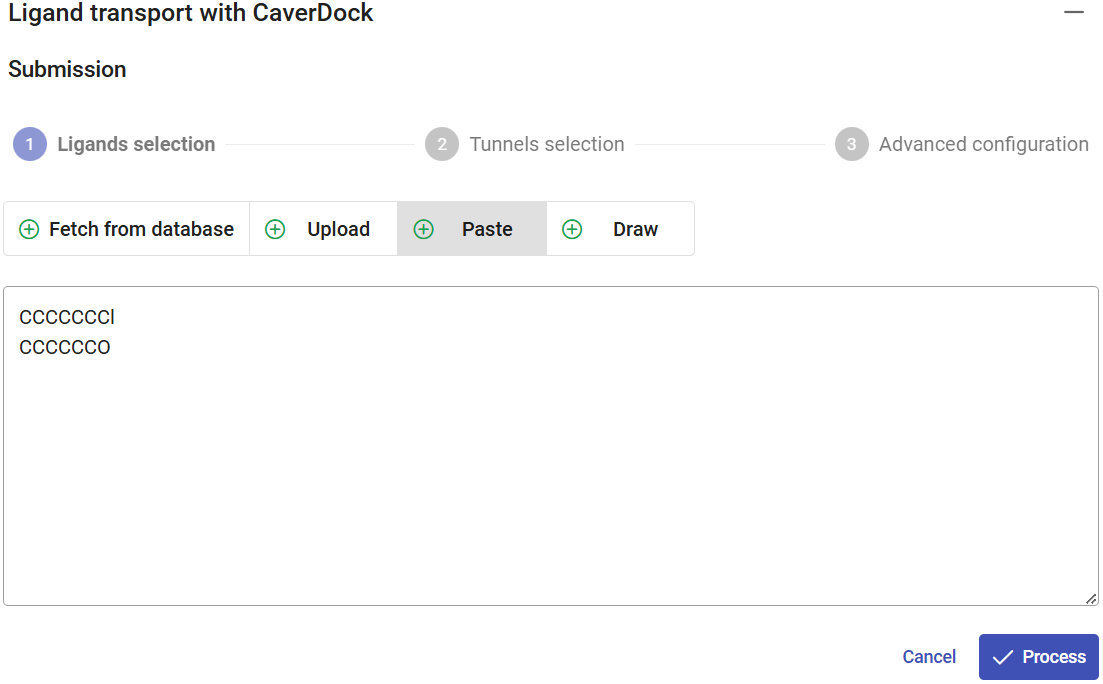


Rename the ligands accordingly. In this case, only the “out” direction is allowed. The reason is that, due to the protein flexibility, the tunnels may vary in length. Therefore, the distance travelled by the ligands going inwards can be different in every snapshot, thus leading to imprecisions in the distance associated to the CaverDock energy profiles, and consequently, also to errors in their interpretation.


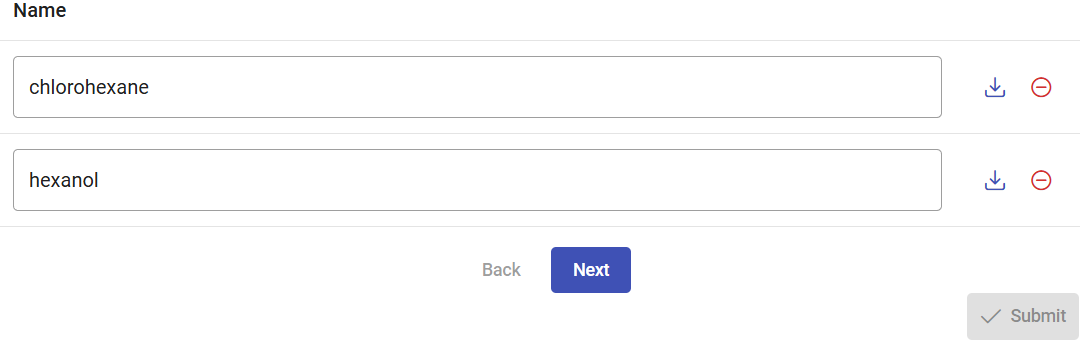


### Selecting the tunnels

In Use Case 1, section 2, we found that tunnel 1 was the most likely used for transport of both substrate and product. Because CaverDock calculations need reasonable computational resources, in dynamic mode you can select only one tunnel in each submission. Therefore, select tunnel 1 for the current calculations, and click on “Next”.


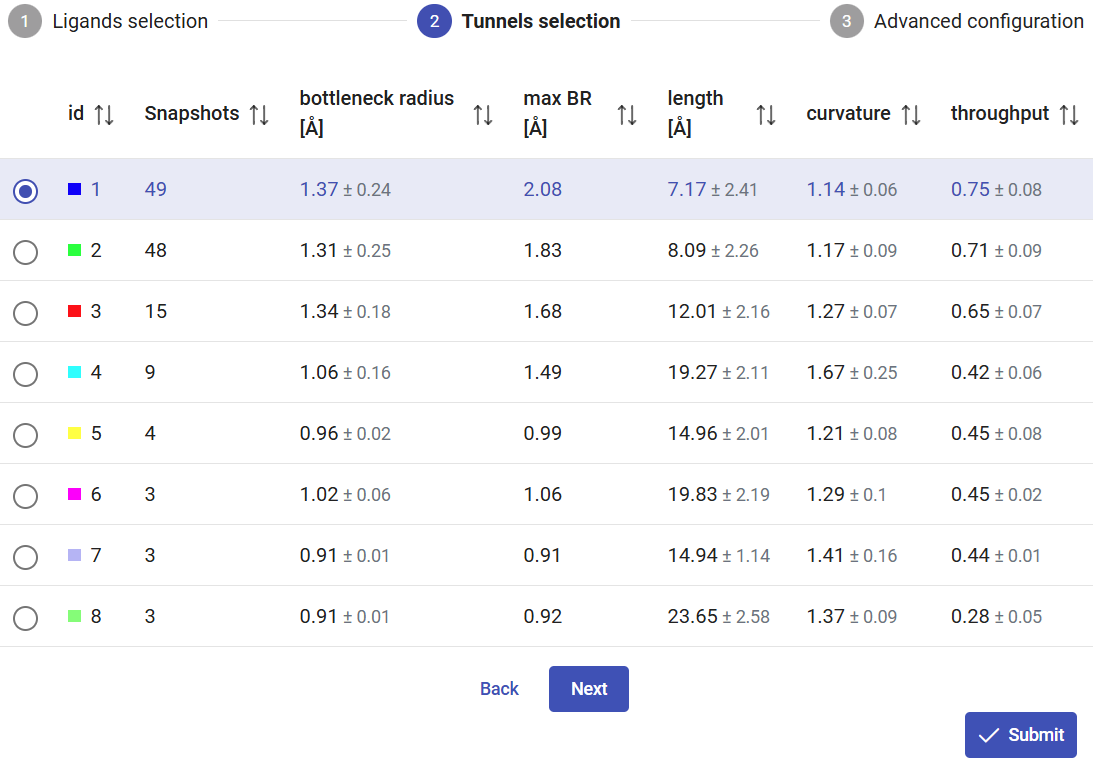


### Setting up advanced parameters

As described in Use Case 1, section 2 set the CL7 atom of chlorohexane and O7 atom of hexanol as the drag atoms. These atoms, in the respective ligands, will be placed in each sequential step of the CaverDock calculation to obtain the trajectory and binding energy profile. Click on “Submit” to start the CaverDock calculations for those two ligands in tunnel 1. These calculations will take a few hours to finish, since CaverDock will be run for each ligand in every snapshot where the tunnel was identified.


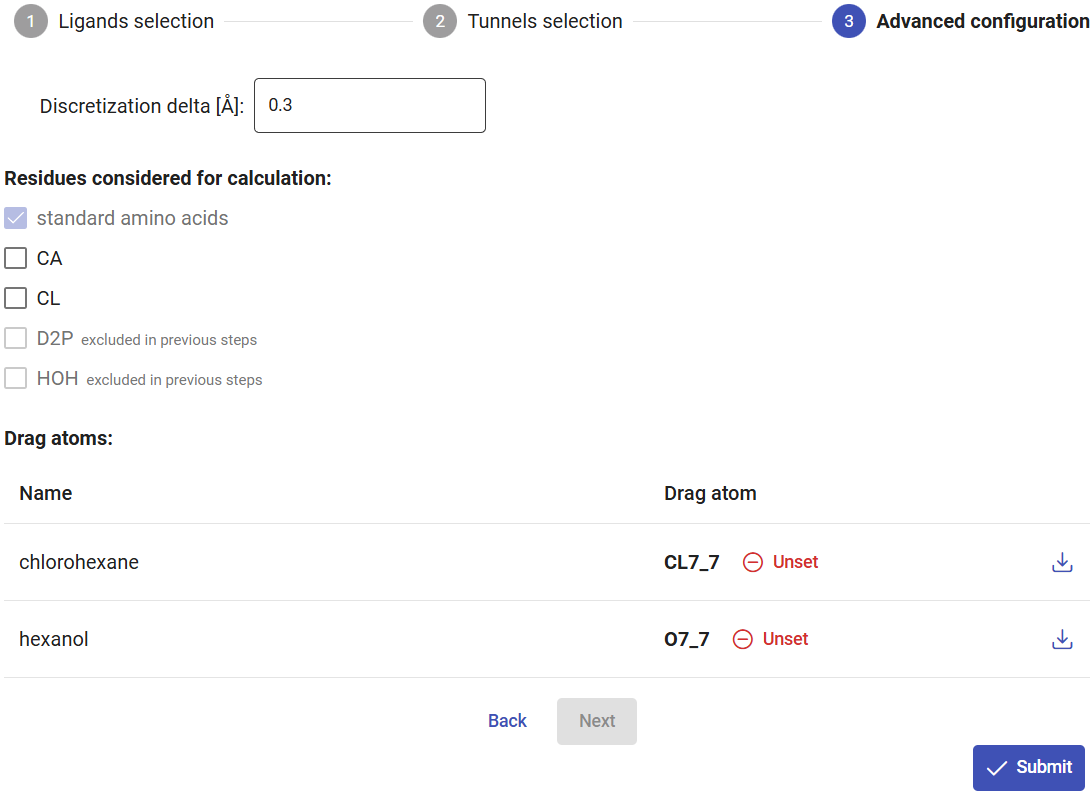


### Analyzing the results

After the CaverDock jobs have finished, they can be found in the Results panel (which lists all the CaverDock jobs) with a green check mark on the left if they finished successfully. Each job is named by the ligand name that you defined before, indicates the dragged atom (if any), the selected tunnel for the CaverDock calculation.

At the right-end of each job you can find two icons. The “Download raw data in zip archive” (
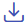
) to save the original calculation folder. If you click on “Show results” (
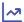
 ), it will display the CaverDock energy profiles, which shows the change in the binding energy of the ligand moving along the tunnel out of the active site to the surface. Therefore, the “surface” will be at the left of the graph (Distance 0) and the “active site” at the right. For a more detailed explanation, see Use Case 1, section 2.


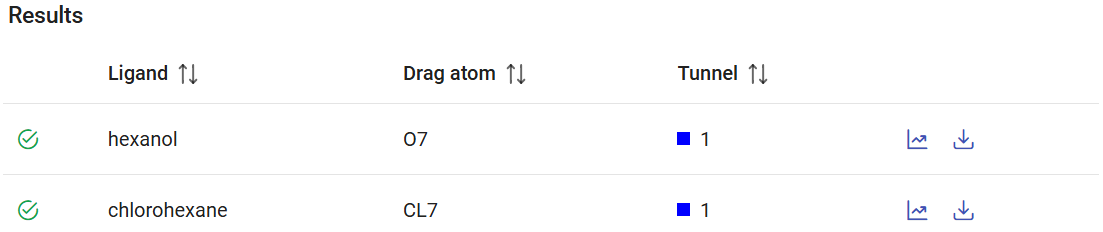


Click on “show results” for the calculation with hexanol in tunnel 1. You will find the CaverDock energy profile for the ligand traveling from the active site to the surface. In this dynamic mode, the profiles are accompanied with the respective standard error, which take into account the CaverDock profiles calculated for all the snapshots in the MD simulation.

Here, we remind you that we recommend specifying the E_surface_ as the minimum-energy near the surface, because often there is a shallow valley at the tunnel mouth where the ligand binds with non-zero energy, or the energy-plateau if there is one. E_max_ should be specified at the maximum energy point along the tunnel. E_bound_ is the minimum-energy value near the binding site, because sometimes the energy can spike when the ligand is forced too close to the bottom of the cavity.

We want to highlight that CaverDock is not a replacement for detailed (un)binding MD simulations or free-energy methods. As an approximate docking-based approach, it is well suited for rapid screening or rough estimation. Although its accuracy improves significantly when multiple protein snapshots are considered (3), the resulting energy profiles remain approximate and are best used for comparative purposes rather than absolute energetic interpretation.

This profile can be compared with the profile obtained from the static structure for the same hexanol ligand in the same tunnel 1 (see Use Case 1, section 2; job [pxtxpx](https://loschmidt.chemi.muni.cz/caverweb/job/pxtxpx/)), and we can conclude that there are significant differences. First of all, now we can assign a confidence interval to the binding energy at each point. We can also observe that the energy maximum became smoother. This is due to the natural fluctuations within the protein, including its tunnels and bottlenecks, and therefore also on the interactions that those residues can form with the ligand. The tunnels also seem longer than they were for a single structure. This is simply because the changes in the protein in some snapshots narrowed down the tunnel mouth, and as a consequence the overall length of the tunnel was extended.


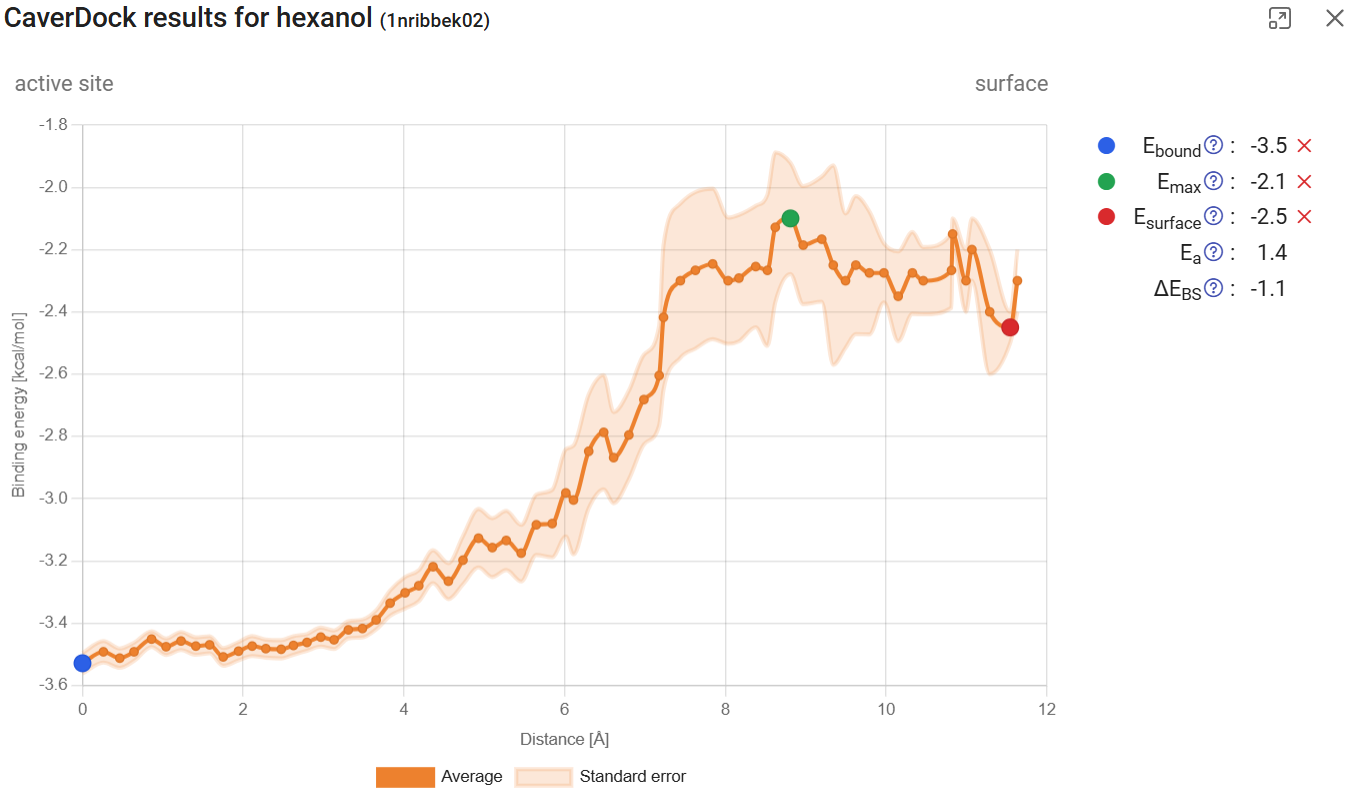


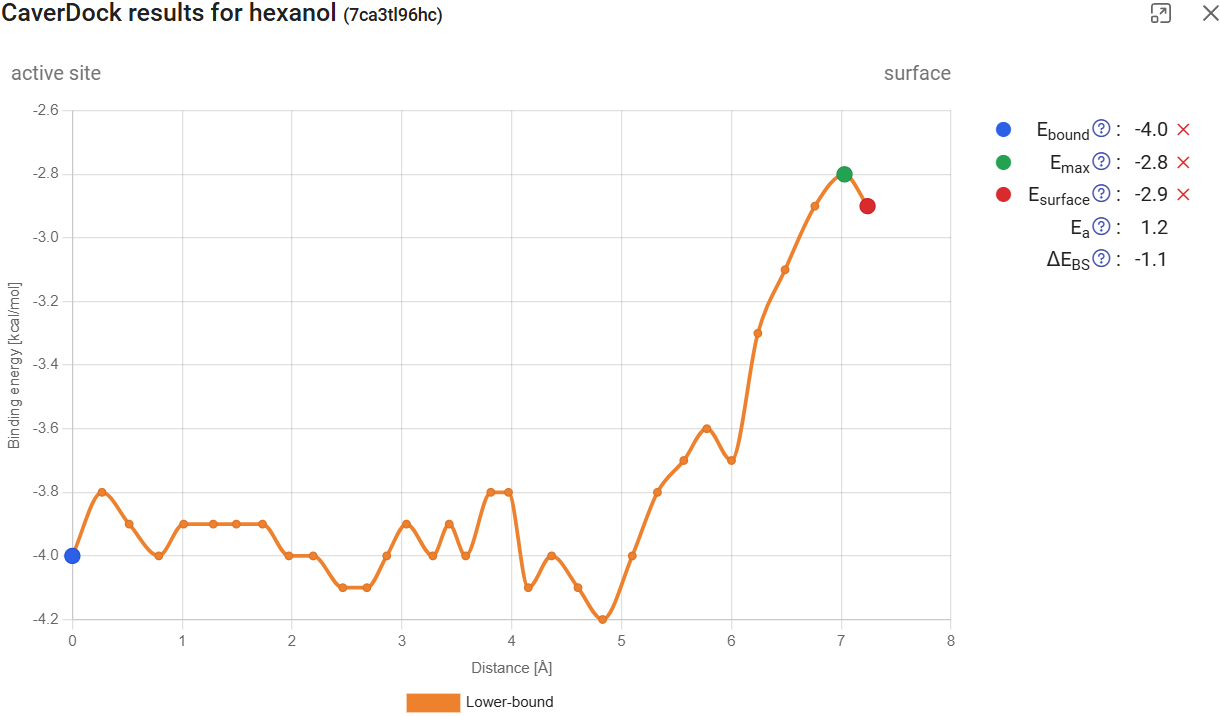


As mentioned above, in dynamic mode only the outwards direction is allowed. This means that the energy profile of substrates (which typically should travel inwards) are inverted, but the binding energies are comparable. This is because, for the lower-bound trajectory, the direction is irrelevant. The resulting profile for chlorohexane, now with a standard error associated to each point of the trajectory, can be compared with the one obtained for a single structure (Use Case 1, section 2; job [pxtxpx](https://loschmidt.chemi.muni.cz/caverweb/job/pxtxpx/)).


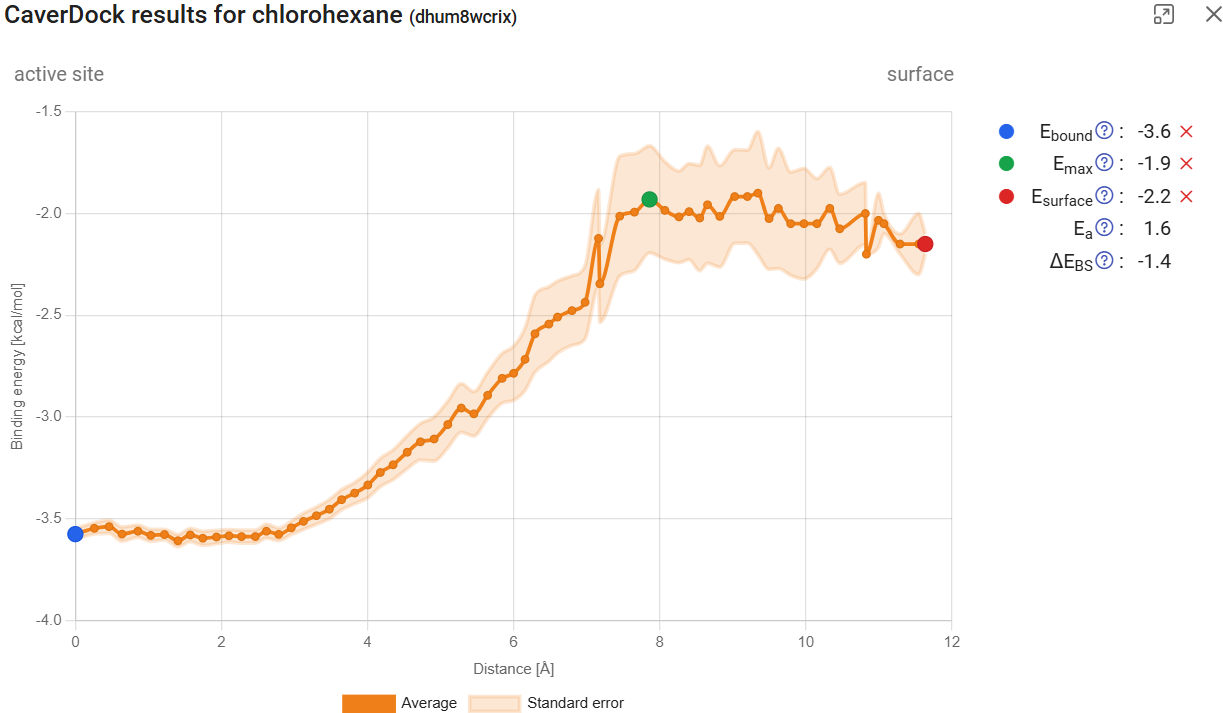


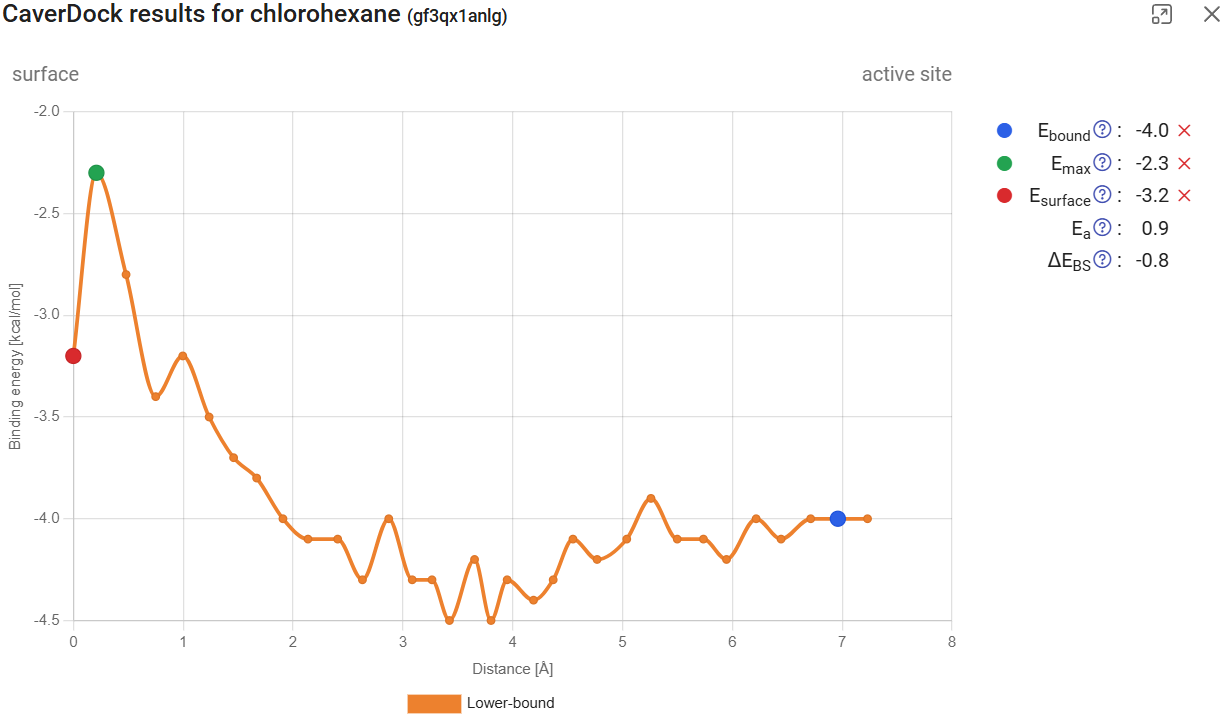


## References

1. Marques,S.M., Dunajova,Z., Prokop,Z., Chaloupkova,R., Brezovsky,J. and Damborsky,J. (2017) Catalytic Cycle of Haloalkane Dehalogenases Toward Unnatural Substrates Explored by Computational Modeling. *J. Chem. Inf. Model.*, **57**, 1970–1989.

https://doi.org/10.1021/acs.jcim.7b00070

2. Kaushik,S., Marques,S.M., Khirsariya,P., Paruch,K., Libichova,L., Brezovsky,J., Prokop,Z., Chaloupkova,R. and Damborsky,J. (2018) Impact of the access tunnel engineering on catalysis is strictly ligand-specific. *FEBS J.*, **285**, 1456–1476.

https://doi.org/10.1111/febs.14418

http://www.ncbi.nlm.nih.gov/pubmed/29478278

3. Vavra,O., Tyzack,J., Haddadi,F., Stourac,J., Damborsky,J., Mazurenko,S., Thornton,J.M. and Bednar,D. (2024) Large-scale annotation of biochemically relevant pockets and tunnels in cognate enzyme–ligand complexes. *Journal of Cheminformatics*, **16**, 114.

https://doi.org/10.1186/s13321-024-00907-z

4. Krieger,E. and Vriend,G. (2014) YASARA View—molecular graphics for all devices—from smartphones to workstations. *Bioinformatics*, **30**, 2981–2982.

https://doi.org/10.1093/bioinformatics/btu426

5. Krieger,E. and Vriend,G. (2015) New ways to boost molecular dynamics simulations. *J Comput Chem*, **36**, 996–1007.

https://doi.org/10.1002/jcc.23899

http://www.ncbi.nlm.nih.gov/pmc/articles/PMC6680170

6. Chovancová,E., Pavelka,A., Benes,P., Strnad,O., Brezovsky,J., Kozlikova,B., Gora,A., Sustr,V., Klvana,M., Medek,P., *et al.* (2012) CAVER 3.0: a tool for the analysis of transport pathways in dynamic protein structures. *PLoS Comput. Biol.*, **8**, e1002708.

https://doi.org/10.1371/journal.pcbi.1002708
